# Composition-Dependent Performance of Hydrophobic Glass Wool Fiber Aerogels for Oil Absorption and Thermal Insulation

**DOI:** 10.3390/gels12090831

**Published:** 2026-09-11

**Authors:** Thi Thanh Hai Dam, Thanh Thanh Le, Nguyen Thi Hong Phuc, Nga H. N. Do, Quang M. N. Phan, Phan Minh Quoc Binh, Hai M. Duong

**Affiliations:** 1 Petroleum Faculty, PetroVietNam University (PVU), 762 Cach Mang Thang Tam Street, Ba Ria Ward, Ho Chi Minh City 78117, Vietnam; haidtt@pvu.edu.vn (T.T.H.D.); thanhlt@pvu.edu.vn (T.T.L.); phucnth@pvu.edu.vn (N.T.H.P.); 2 Institute for Tropical Technology (VITTEP), 57A Truong Quoc Dung Street, Phu Nhuan Ward, Ho Chi Minh City 70073, Vietnam; ngado.spe@gmail.com; 3 Department of Mechanical Engineering, National University of Singapore (NUS), Singapore 117575, Singapore; quang.phanngominh@u.nus.edu; 4 Vietnam Petroleum Institute (VPI), 167 Trung Kinh Street, Yen Hoa Ward, Hanoi 100000, Vietnam; 5 Mekong University (MKU), Vinh Long Province 85216, Vietnam

**Keywords:** glass wool fiber aerogel, methyltrimethoxysilane, crude oil absorption, absorption kinetics, cyclic reusability, thermal insulation

## Abstract

Glass wool provides a lightweight fibrous framework with inherent thermal-insulation capability, yet its direct use in hydrophobic monolithic aerogels has received comparatively limited systematic investigation. Here, glass wool fiber (GWF)/poly(vinyl alcohol) (PVA) aerogels were fabricated by freeze-drying followed by vapor-phase methyltrimethoxysilane (MTMS) modification. A composition matrix of 1.0–3.0 wt.% GWF and 0.10–1.00 wt.% PVA was evaluated to determine composition-dependent changes in density, calculated porosity, wettability, compressive response, thermal conductivity, crude-oil absorption, uptake kinetics, and cyclic reusability. The aerogels exhibited densities of 0.014–0.046 g/cm^3^, calculated porosities of 97.68–99.39%, water contact angles of 131.0–141.3°, thermal conductivities of 32.1–38.5 mW/m·K, and compressive stress at 50% strain up to 146.20 kPa. Crude-oil absorption, defined here as predominantly physical uptake and retention within the porous fibrous network, ranged from 18.86 ± 1.50 to 55.12 ± 1.57 g/g. At 0.25 wt.% PVA, samples containing 1.0–3.0 wt.% GWF reached 81–91% of equilibrium uptake within 10 s. The pseudo-second-order model provided the better empirical fit without implying chemisorption. Overall, composition influenced the balance among oil uptake, mechanical resistance, cyclic reuse, and thermal insulation.

## 1. Introduction

Oil pollution remains a major threat to aquatic ecosystems because of its persistence, mobility, and difficulty of remediation [1,2,3,4]. Conventional responses to oil spills and oily wastewater include chemical, biological, and physical approaches; however, each has practical limitations. Chemical dispersants and in situ burning can rapidly reduce floating oil but may introduce secondary environmental impacts [5,6], whereas biological treatments are generally slower and strongly dependent on environmental conditions [7]. In comparison, physical oil removal using porous sorbent materials offers operational simplicity, recoverability, and the possibility of material reuse [8,9,10]. Consequently, considerable attention has been directed toward low-density sorbents that can float on water, exhibit hydrophobic and oleophilic behavior, rapidly take up oil, and retain sufficient structural integrity for recovery and reuse [11,12].

Aerogels are attractive candidates for oil-remediation applications because their low density and high void fraction provide substantial space for liquid uptake and retention [13,14]. Hydrophobic fiber-based aerogels derived from biomass, recycled polymers, and other fibrous waste streams have been widely investigated for oil–water separation and oil-spill remediation [13,15]. Their performance is governed not only by overall porosity but also by surface wettability, fiber-network connectivity, accessible void space, and structural integrity. Beyond static wettability, dynamic liquid–surface interactions can depend strongly on interfacial and operating conditions, as demonstrated in a recent study of droplet dynamics on superhydrophobic surfaces [16]. Accordingly, in the present context, static water contact angle (WCA) measurements are used primarily as indicators of water repellency rather than as direct descriptors of liquid transport through the porous network. Selected fiber-based aerogels have also demonstrated combined oil-removal and thermal-insulation functions [17], highlighting the potential of lightweight porous materials to integrate environmental remediation with thermal functionality. However, increasing the fiber or binder content to strengthen the porous framework may increase the solid fraction and modify the available void space and transport pathways, potentially leading to trade-offs among mechanical resistance, liquid uptake, and thermal performance [15,18].

Recent developments in advanced porous and architected materials further indicate that functional performance cannot be interpreted solely from bulk porosity. Network topology, hierarchical organization, and the connectivity of the load-bearing framework can strongly influence load transfer, deformation, and transport behavior [19,20]. For example, Xie et al. demonstrated that a sponge-bone-like reticular nanoscale architecture can substantially modify the mechanical response of a lightweight structural material, while Shen et al. showed that hierarchical, self-similar topology in a bio-inspired re-entrant honeycomb markedly enhances specific stiffness and energy absorption [19,20]. Although these systems differ fundamentally in composition and fabrication from fibrous aerogels, they illustrate the broader principle that the organization and connectivity of a lightweight network can be as important as its overall solid fraction. More directly relevant to aerogel-based multifunctional materials, Zhang et al. integrated composite aerogels with architected mechanical metamaterials to combine load-bearing capability with thermal-insulation, acoustic, and electromagnetic functions [21]. These advances motivate the evaluation of porous aerogels in terms of composition-dependent structural and functional trade-offs rather than attributing performance solely to high porosity.

From a mechanical-durability perspective, the response of lightweight and heterogeneous materials under repeated or dynamic deformation cannot be inferred solely from a single monotonic strength value. Beyond the influence of structural organization on stiffness and energy absorption demonstrated in mechanically architected cellular systems [20], the dynamic compression studies of fiber-containing composites have highlighted the importance of damage evolution and energy dissipation under high-rate loading conditions [22]. Although these material systems differ substantially from fibrous GWF/PVA aerogels, they provide useful broader mechanical frameworks and emphasize the need to distinguish initial compressive resistance from cyclic or long-term mechanical durability. This distinction is particularly relevant to reusable oil sorbents, for which mechanical oil recovery by squeezing subjects the material to repeated deformation and requires sufficient structural integrity over successive absorption–recovery cycles.

Mineral fibrous materials, particularly glass wool, are attractive starting frameworks for lightweight porous materials because of their low density, commercial availability, and well-established thermal-insulation capability [23]. Glass wool inherently consists of randomly oriented inorganic fibers forming an open three-dimensional network, and its multiscale fibrous architecture has been investigated using techniques including X-ray micro-computed tomography [24]. Previous studies have employed glass wool in three main ways for porous-material and oil–water-separation applications. First, glass-fiber felts have been used as reinforcing scaffolds for silica aerogel composites to improve mechanical and dimensional stability [25,26]. Second, glass wool or glass micro-fiber substrates have been directly functionalized with low-surface-energy coatings for oil–water separation [27,28]. Third, mineral wool has been used as a silica source, requiring the dissolution of the original fibers before subsequent aerogel formation [29]. These approaches demonstrate the versatility of glass wool but either introduce a distinct aerogel phase, focus primarily on surface-functionalized fibrous substrates, or remove the original glass wool architecture during precursor extraction. The direct use of the pre-existing glass wool network as the principal structural framework of a monolithic hydrophobic aerogel has therefore received comparatively limited systematic investigation [25,26,27,28,29].

Poly(vinyl alcohol) (PVA) and methyltrimethoxysilane (MTMS) provide complementary functions for converting fibrous frameworks into mechanically coherent and water-repellent aerogels. PVA has been widely employed as a water-processable binder in fiber-based aerogels, where its hydroxyl-rich polymer chains promote inter-fiber cohesion and help preserve monolithic integrity after drying [15,30,31,32]. MTMS was selected for surface modification because its hydrolysable methoxy groups can form silanol-containing species and subsequently undergo condensation, whereas its methyl substituent introduces low-surface-energy groups that promote hydrophobicity [31,32,33]. Vapor-phase MTMS treatment has been successfully applied to highly porous fiber-based aerogels, providing hydrophobic surfaces while retaining the underlying porous fibrous framework [30,31,32]. Importantly, thermal conductivity was investigated here not as an unrelated secondary property, but to determine whether the intrinsic thermal-insulation functionality of the glass wool framework could be retained after PVA incorporation, freeze-drying, and hydrophobic surface modification [23]. This rationale is also consistent with recent developments in multifunctional lightweight porous materials and thermal-management systems that seek to integrate structural integrity, thermal transport, and multifunctional performance within a single architecture [21,34,35].

Against this background, the key question addressed in the present work is how the relative contents of glass wool fiber (GWF) and PVA influence the balance between measured physical characteristics and functional performance in MTMS-modified GWF/PVA aerogels. Rather than attributing performance solely to high porosity, the present study evaluates composition-dependent trends using bulk density, calculated porosity, morphology observed by scanning electron microscopy (SEM), wettability, compressive response, thermal conductivity, crude-oil absorption, uptake kinetics, and cyclic absorption-recovery behavior, consistent with the broader view that network organization and composition jointly influence multifunctional performance in lightweight porous materials [19,20,21]. GWF/PVA aerogels containing 1.0–3.0 wt.% GWF and 0.10–1.00 wt.% PVA were fabricated by freeze-drying followed by vapor-phase MTMS modification. The objective was to establish how fiber and binder contents govern the experimentally observed trade-offs among oil uptake, mechanical resistance, cyclic reusability, and thermal insulation, while preserving the intrinsic glass wool network as the principal porous framework.

## 2. Results and Discussion

### 2.1. Morphology and Physicochemical Characteristics of Glass Wool Fiber Aerogels

**Figure 1:**
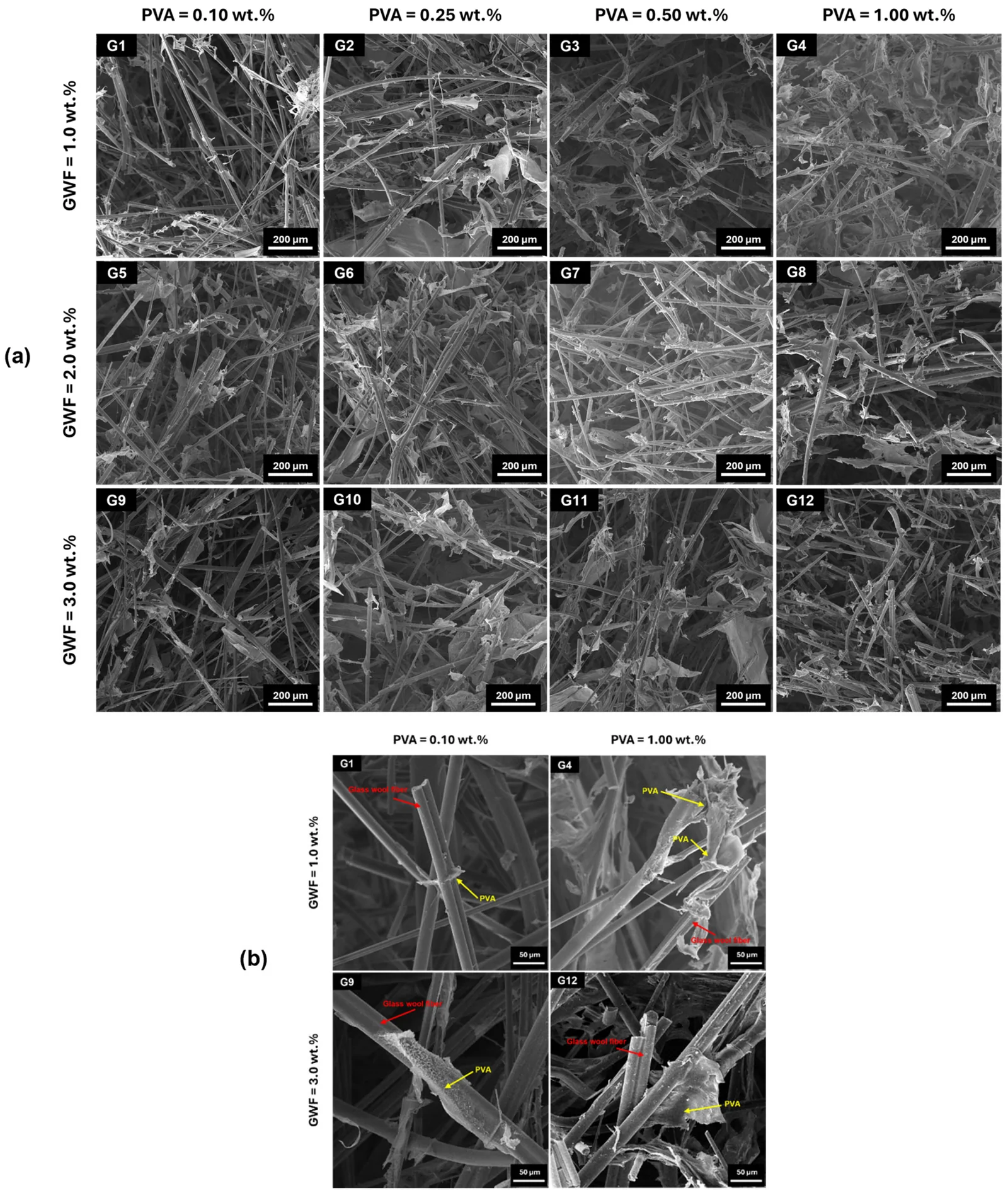
Morphological characteristics of GWF aerogels. (**a**) SEM micrographs of GWF aerogels G1–G12 prepared with varying GWF contents and PVA concentrations at 250× magnification (scale bars: 200 μm). (**b**) Higher-magnification SEM images of the four corner formulations of the experimental matrix (G1, G4, G9, and G12) at 1000× magnification (scale bars: 50 μm).

The SEM images at a magnification of 250× for the twelve formulations (G1–G12) show that the fibrous framework was well-preserved after the freezing and freeze-drying processes (Figure 1). Throughout the observed fields, the GWFs exhibited an elongated morphology and were randomly oriented, interwoven, and overlapped at multiple contact points. This interwoven fibrous framework served as the primary structural framework of the aerogel, whereas the spaces between adjacent fibers appeared as irregular inter-fiber voids with non-uniform apparent sizes and shapes.

This observation is consistent with previous studies on aerogels based on bagasse fibers and waste wool fibers, in which the preservation of the fibrous network after freeze-drying contributed to the formation of lightweight, highly porous materials with excellent potential for oil absorption [17,36]. More broadly, the importance of reinforcement architecture and orientation in governing the integrated mechanical and functional performance of composite materials has also been demonstrated in aligned CNT/epoxy systems [37].

The SEM images acquired at a magnification of 1000× provide a more detailed view of the fiber surfaces, fiber–fiber contact regions, and localized film-like and bridge-like structures (Figure 1b). In G1, which contained 1.0 wt.% GWF and 0.10 wt.% PVA, the fibers remained largely discrete, with only a small bridge-like feature present at the junction between two adjacent fibers. As the PVA concentration increased, film-like and flake-like deposits, together with inter-fiber bridges, became increasingly prominent. In G4 and G12, these structures partially coated the fiber surfaces and extended across neighboring fibers, indicating more extensive inter-fiber connectivity and suggesting enhanced cohesion within the fibrous network. A localized film-like coating was also present on the fiber surface in G9, although it was less continuous and extensive than that in G12.

Considering the initial composition of the system and the role of PVA during aerogel fabrication, the observed film-like and bridge-like features are assigned to PVA-rich binder regions retained within the aerogel framework. PVA likely adhered to the fiber surfaces and accumulated at fiber–fiber contact points, thereby promoting inter-fiber connectivity and helping preserve the structural integrity of the monolithic aerogel. The use of PVA as a binder has also been widely reported in various fiber-based aerogels [17,30,31,32,36].

As summarized in Table 1, the GWF aerogels had very low bulk densities, ranging from 0.014 to 0.046 g/cm^3^, and correspondingly high calculated porosities of 97.68–99.39%. Among the samples, G1, which contained 1.0 wt.% GWF and 0.10 wt.% PVA, showed the lowest bulk density of 0.014 ± 0.001 g/cm^3^ and the highest porosity of 99.39 ± 0.03%. Conversely, G12, containing 3.0 wt.% GWF and 1.00 wt.% PVA, had the highest bulk density of 0.046 ± 0.004 g/cm^3^ and the lowest porosity of 97.68 ± 0.21%. Despite the substantial differences in solid content among the formulations, all aerogels retained calculated porosities above 97%, indicating highly porous structures with large void fractions. These values are comparable to those reported for other PVA-bonded fiber-based aerogels, such as bagasse/PVA aerogels (91.9–98.9%) and wool fiber/PVA aerogels (97.73–99.63%) [17,36]. The consistently high calculated porosity values, together with the open fibrous morphology observed by SEM, suggest that the freeze-drying process and PVA-mediated fiber bonding preserved a lightweight structure with a high bulk void fraction.

The influence of PVA concentration was consistent across all investigated GWF content levels. For formulations containing 1.0 wt.% GWF, increasing the PVA concentration from 0.10 to 1.00 wt.% increased the bulk density from 0.014 to 0.025 g/cm^3^ and reduced the calculated porosity from 99.39% to 98.42% (G1–G4). A similar trend was observed at 2.0 wt.% GWF, with the bulk density increasing from 0.024 to 0.034 g/cm^3^ and the calculated porosity decreasing from 98.97% to 98.17% (G5–G8). At 3.0 wt.% GWF, the bulk density likewise increased from 0.036 to 0.046 g/cm^3^, whereas the calculated porosity declined from 98.49% to 97.68% (G9–G12). Overall, increasing the PVA concentration produced denser aerogel structures while causing only a modest reduction in calculated porosity.

The observed increase in bulk density and decrease in calculated porosity with increasing PVA concentration may be attributed to the greater amount of polymer retained within the aerogel network after freeze-drying, thereby increasing the solid fraction. PVA may also have accumulated on the fiber surfaces and at fiber–fiber contact regions, forming localized film-like and bridge-like features. These binder-rich regions could promote inter-fiber cohesion and help maintain the structural integrity of the monolithic aerogel while locally narrowing some of the inter-fiber openings visible in the SEM images. This interpretation is qualitatively supported by the more prominent film-like and bridge-like features observed in the higher-PVA formulations, particularly G4 and G12 (Figure 1b). Nevertheless, the SEM micrographs represent only selected areas of the samples and are therefore insufficient to establish a monotonic increase in either the abundance or surface coverage of these features throughout the entire aerogel structure. The role of PVA as a binder has also been widely reported in various fiber-based aerogel systems [17,30,31,32,36].

In addition to the effect of PVA concentration, Table 1 shows that increasing the GWF content also increased the bulk density and reduced the calculated porosity. At a fixed PVA concentration of 0.25 wt.%, increasing the GWF content from 1.0 to 3.0 wt.% in the G2–G6–G10 series increased the bulk density from 0.016 to 0.038 g/cm^3^ and correspondingly decreased the calculated porosity from 99.21% to 98.34%. Similar trends were observed for the G1–G5–G9, G3–G7–G11, and G4–G8–G12 series. This behavior is primarily attributed to the greater amount of fibrous solid per unit volume at higher GWF contents. The resulting denser fibrous framework may also increase fiber–fiber overlap and contact, consistent with the lower calculated bulk porosity observed at higher GWF contents. Nevertheless, all formulations retained calculated porosities above 97%, indicating that a high bulk void fraction was maintained across the investigated GWF content range.

It should be emphasized that the porosity values reported here were calculated from bulk density and constituent densities and therefore represent estimates of the overall bulk void fraction rather than direct measurements of pore-size distribution or liquid-accessible pore volume. The SEM images qualitatively reveal open inter-fiber spaces, fiber–fiber contacts, and localized PVA-rich features; however, two-dimensional micrographs cannot quantify three-dimensional pore connectivity, pore-volume distribution, or the fraction of void space accessible to oil. Recent studies on architected porous materials have demonstrated the value of quantitative structural metrics for establishing rigorous structure–property relationships [19,21], while X-ray micro-computed tomography has specifically been used to resolve the multiscale fibrous architecture of glass wool [24]. Accordingly, the structure–property interpretations in the present study are treated as qualitative correlations based on bulk density, calculated porosity, and SEM-observed morphology rather than as direct measurements of the three-dimensional pore network.

Overall, the SEM observations, bulk-density measurements, and calculated porosity values indicate that the GWF aerogels retained an open, interwoven fibrous morphology together with a high calculated bulk void fraction across the investigated composition range.

The SEM analysis in this study was designed to compare the morphology of the aerogel formulations and to support the interpretation of composition-dependent properties, rather than to track morphological evolution at individual fabrication and MTMS modification stages. Accordingly, the SEM observations are not used as evidence of morphological changes specifically induced by MTMS treatment.

**Figure 2:**
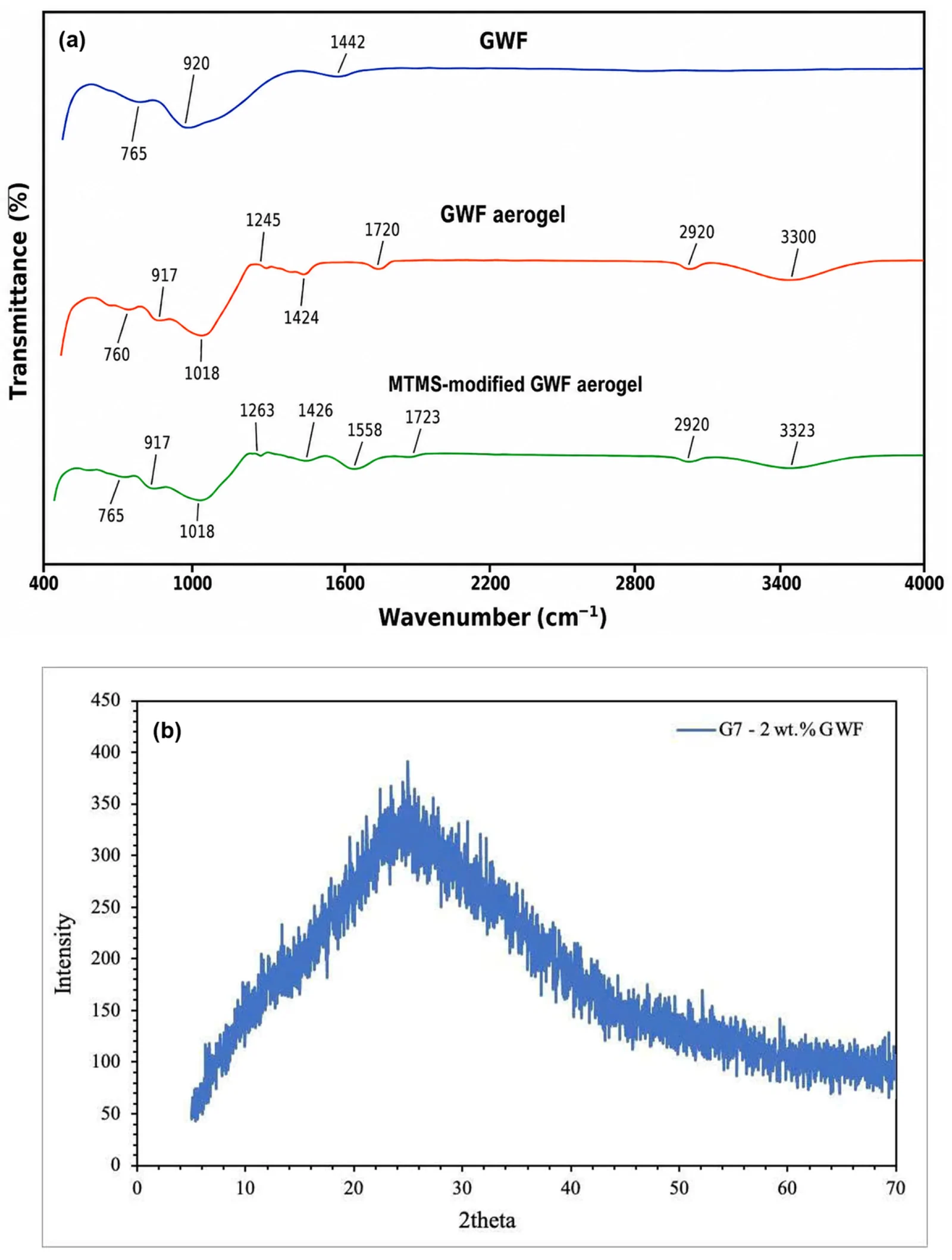
Chemical and structural characterization of GWF aerogels. (**a**) FTIR spectra of pristine GWF, unmodified GWF aerogel, and MTMS-modified GWF aerogel. (**b**) XRD pattern of the representative GWF aerogel G7 containing 2.0 wt.% GWF and 0.50 wt.% PVA.

The chemical and structural characteristics associated with aerogel fabrication and subsequent surface modification were further examined by Fourier-transform infrared (FTIR) spectroscopy and X-ray diffraction (XRD) (Figure 2). Figure 2a compares the FTIR spectra of pristine GWF, the unmodified GWF aerogel, and the MTMS-modified GWF aerogel. Pristine GWF exhibited a broad absorption envelope between approximately 1200 and 800 cm^−1^, attributable primarily to Si–O stretching vibrations within the silicate network. Prominent bands near 920 and 765 cm^−1^ were associated mainly with the native silicate structure, whereas a weaker band near 1442 cm^−1^ was also observed [33].

After PVA incorporation and freeze-drying, the GWF aerogel exhibited a broad absorption band near 3300 cm^−1^ and an additional band near 2920 cm^−1^. The broad band near 3300 cm^−1^ is attributed to overlapping O–H stretching contributions from PVA, surface silanol groups, and adsorbed moisture [38,39], whereas the band near 2920 cm^−1^ is assigned to the aliphatic C–H stretching of PVA [38]. A feature near 1424 cm^−1^ may include a contribution from the CH_2_ bending vibration of PVA [38]; however, because pristine GWF showed a nearby band at approximately 1442 cm^−1^, this feature should not be regarded as arising exclusively from PVA. A weak band near 1720 cm^−1^ may be associated with the C=O stretching vibration of residual acetate groups in PVA. Because this assignment depends on the degree of PVA hydrolysis, it should be regarded as tentative [38]. A weak shoulder was also observed near 1245 cm^−1^; however, owing to spectral overlap in this region, no definitive assignment was made. The features near 1018, 917, and 760 cm^−1^ remained within the characteristic low-wavenumber region of the glass wool silicate network, supporting the preservation of the native GWF framework after PVA incorporation and freeze-drying. Collectively, the appearance of the bands near 3300 and 2920 cm^−1^, together with the spectral changes observed in the intermediate-wavenumber region, supports the incorporation and retention of PVA within the dried aerogel structure, consistent with its proposed role as a binder between the GWFs [36,38].

**Figure 3:**
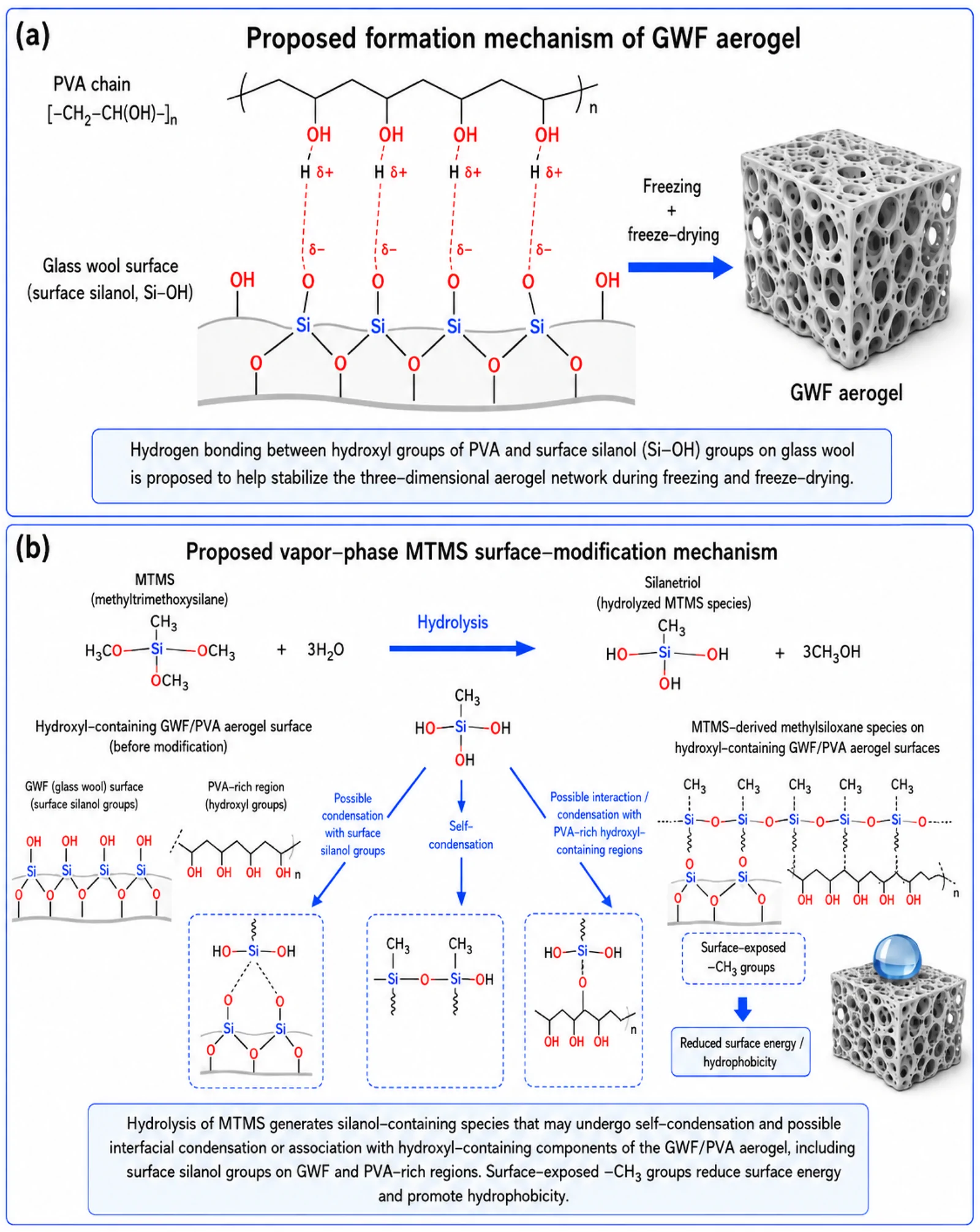
Schematic illustration of the proposed mechanisms for (**a**) PVA-mediated stabilization of the GWF aerogel network and (**b**) possible pathways of vapor-phase MTMS surface modification.

Following MTMS treatment, the principal features observed in the unmodified GWF aerogel remained evident at approximately 3323, 2920, 1723, and 1426 cm^−1^. Notably, a feature near 1263 cm^−1^ became more clearly resolved in the modified aerogel, within a region where the unmodified aerogel exhibited a weak shoulder near 1245 cm^−1^. This feature is associated with Si–CH_3_-related vibrations [39] and provides spectroscopic evidence consistent with the presence of methyl-containing siloxane species following MTMS treatment [39,40,41]. The development of this feature is also consistent with the proposed MTMS modification pathways illustrated in Figure 3. A weak feature was also observed near 1558 cm^−1^; however, owing to its low intensity and possible spectral overlap, no definitive assignment was made.

The strong absorption band centered at approximately 1018 cm^−1^ lies within the broad Si–O stretching region of the glass wool network and may also contain an overlapping contribution from C–O stretching vibrations of PVA [33,38]. Consequently, this band alone cannot be considered conclusive evidence for the formation of new Si–O–Si linkages. Likewise, the bands near 917 and 765 cm^−1^ closely match those observed in pristine GWF at approximately 920 and 765 cm^−1^, respectively, and are therefore assigned mainly to the native silicate network. Nevertheless, overlapping contributions from MTMS-derived vibrations in this spectral region cannot be entirely excluded [33,40].

Overall, the more clearly resolved Si–CH_3_-related feature near 1263 cm^−1^ provides the strongest spectroscopic evidence consistent with the presence of methyl-containing siloxane species on the aerogel surface [39]. The changes observed within the Si–O stretching region are compatible with, but do not independently confirm, the formation of MTMS-derived siloxane structures. Collectively, these spectral features support the presence of methyl-containing species on the GWF aerogel surface following MTMS treatment, consistent with previous reports on MTMS-modified fiber-based aerogels [39,40,41].

The X-ray diffraction (XRD) pattern of the GWF aerogel (sample G7, containing 2.0 wt.% GWF and 0.50 wt.% PVA) is shown in Figure 2b. Over the displayed 2θ range, the pattern is dominated by a broad diffuse halo extending from approximately 8° to 45°, with an apparent maximum near 25.0°. This feature indicates that the material is predominantly amorphous, reflecting the absence of long-range structural order and the glassy nature of the glass wool fibers that form the primary framework of the aerogel [42,43]. No well-defined Bragg reflections were resolved above the background within the displayed range, indicating that no distinct crystalline phase was detectable under the measurement conditions.

The proposed mechanisms underlying GWF aerogel formation and subsequent vapor-phase MTMS modification are schematically illustrated in Figure 3. During aerogel fabrication, hydroxyl groups of PVA may interact with surface silanol groups (Si–OH) on the glass wool fibers through hydrogen bonding, thereby promoting PVA–fiber adhesion and helping to stabilize the three-dimensional fibrous framework during freezing and subsequent freeze-drying [44]. During vapor-phase MTMS treatment, MTMS may undergo hydrolysis in the presence of residual or adsorbed moisture to form silanol-containing species, including methylsilanetriol, CH_3_Si(OH)_3_, which may subsequently self-condense to form methyl-containing siloxane oligomers or networks [45,46]. These hydrolyzed species may also undergo interfacial association and/or possible condensation with hydroxyl-containing components of the GWF/PVA aerogel, including silanol-rich regions of the glass wool surface and PVA-rich regions [32,33,41]. Accordingly, the mechanism illustrated in Figure 3b represents plausible, non-selective surface-modification pathways rather than a unique chemical reaction between MTMS and the glass fibers. Surface-exposed Si–CH_3_ groups are expected to lower the surface energy and thereby promote hydrophobicity [32,41,46]. The Si–CH_3_-related FTIR feature near 1263 cm^−1^ supports the presence of MTMS-derived methyl-containing species; however, the available spectroscopic data do not establish which interfacial modification pathway predominates [46].

For raw GWF and the unmodified GWF/PVA aerogel, a stable apparent WCA could not be determined because the deposited water droplets were rapidly imbibed into the porous fibrous structure. In contrast, all MTMS-modified GWF aerogels supported stable water droplets and exhibited apparent WCA values ranging from 131.0° to 141.3° (Table 1). Together with the Si–CH_3_-related FTIR feature near 1263 cm^−1^, this transition from rapid water imbibition to stable high apparent WCAs supports the introduction of methyl-containing surface species following MTMS treatment. Among the investigated formulations, G9, containing 3.0 wt.% GWF and 0.10 wt.% PVA, exhibited the highest mean WCA of 141.3 ± 1.61°, whereas G8, containing 2.0 wt.% GWF and 1.00 wt.% PVA, showed the lowest value of 131.0 ± 0.61°. Despite this composition-dependent variation, all MTMS-modified samples exhibited apparent WCAs above 130°, confirming pronounced water repellency after vapor-phase modification.

**Figure 4:**
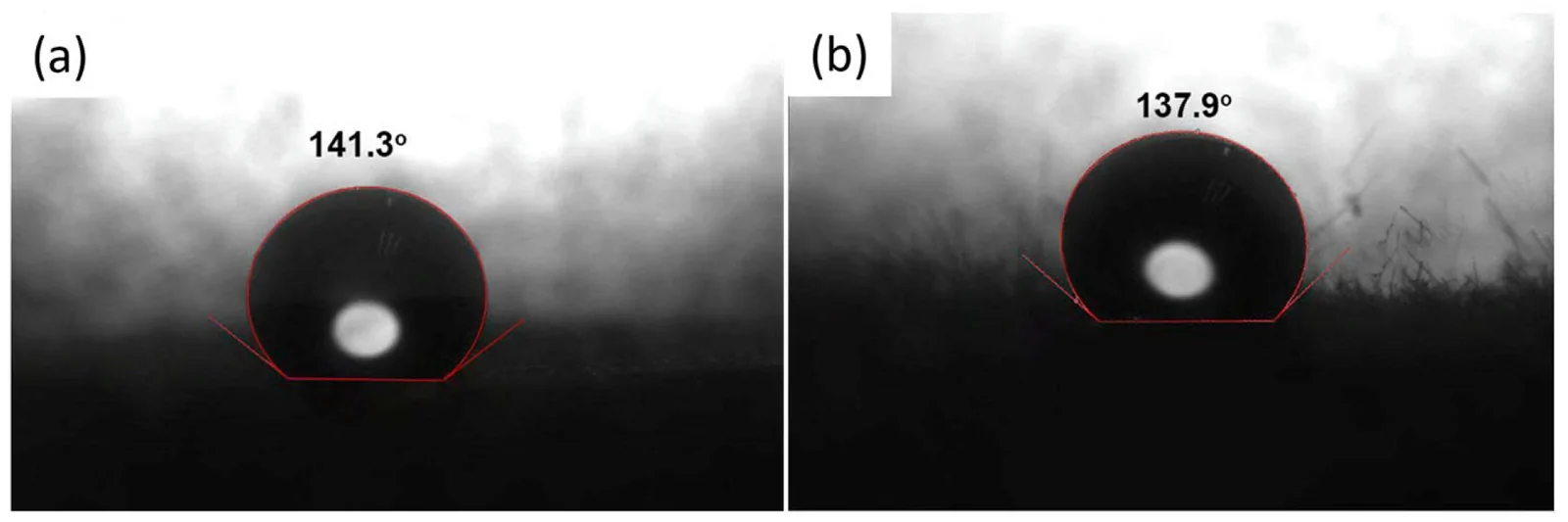
Water contact angles measured on (**a**) the external surface and (**b**) the cross-sectional surface of the MTMS-modified GWF aerogel G9.

For the representative MTMS-modified G9 aerogel, the apparent WCAs measured on the external and cross-sectional surfaces were 141.3° and 137.9°, respectively (Figure 4a,b). The comparable values indicate strong water repellency on both surfaces and are consistent with MTMS modification reaching accessible interior regions of the porous aerogel. However, these measurements do not establish uniform MTMS coverage throughout the entire three-dimensional network. The slightly higher WCA measured on the external surface may reflect its more direct exposure to MTMS vapor during treatment, whereas vapor transport into the internal porous network may have been comparatively restricted; nevertheless, given the small difference between the two values, this interpretation remains tentative. Similar hydrophobic behavior on external and internal regions has been reported for vapor-phase MTMS-modified PVA-containing fibrous aerogels [32,41].

It should be noted that the static WCA measurements in this study were used specifically to assess water repellency and were not interpreted as quantitative descriptors of liquid transport within the porous network. The possible contribution of capillary-assisted transport to crude-oil uptake is discussed separately in Section 2.3.3.

Overall, the combined SEM, bulk-density, calculated porosity, water-contact-angle, FTIR, and XRD results indicate that the fabricated GWF aerogels retained an open, interwoven fibrous morphology with high calculated bulk void fractions. Increasing either the GWF content or the PVA concentration generally increased the solid fraction and bulk density while reducing the calculated porosity, although all formulations retained calculated porosities above 97%. FTIR analysis supports the retention of PVA within the aerogel network and the presence of MTMS-derived methylsiloxane species in the modified aerogel, whereas the XRD pattern of G7 indicates a predominantly amorphous structure. Together with water contact angles above 130°, these findings support the effectiveness of vapor-phase MTMS treatment in imparting pronounced water repellency while preserving the highly porous glass wool framework. The SEM-observed fiber–fiber contacts, solid fraction, and proposed PVA-mediated inter-fiber interactions provide a qualitative structural basis for interpreting the mechanical response discussed in the next section, as well as the oil absorption and thermal behavior examined subsequently.

Statistical analysis further supported these composition-dependent trends. Two-way analysis of variance (ANOVA) showed significant effects of both GWF and PVA contents on bulk density and calculated porosity (all p<0.001). The GWF × PVA interaction was not significant for bulk density ($F\left(624\right)=2.38,\;p=0.060$) but was significant for calculated porosity ($F\left(624\right)=2.69,\;p=0.038$). For WCA, significant effects of GWF content, PVA content, and their interaction were also observed (all p<0.001). These results quantitatively confirm that the measured physical properties were governed by the combined effects of formulation composition rather than by a single compositional variable.

### 2.2. Compressive Behavior of Glass Wool Fiber Aerogels

**Figure 5:**
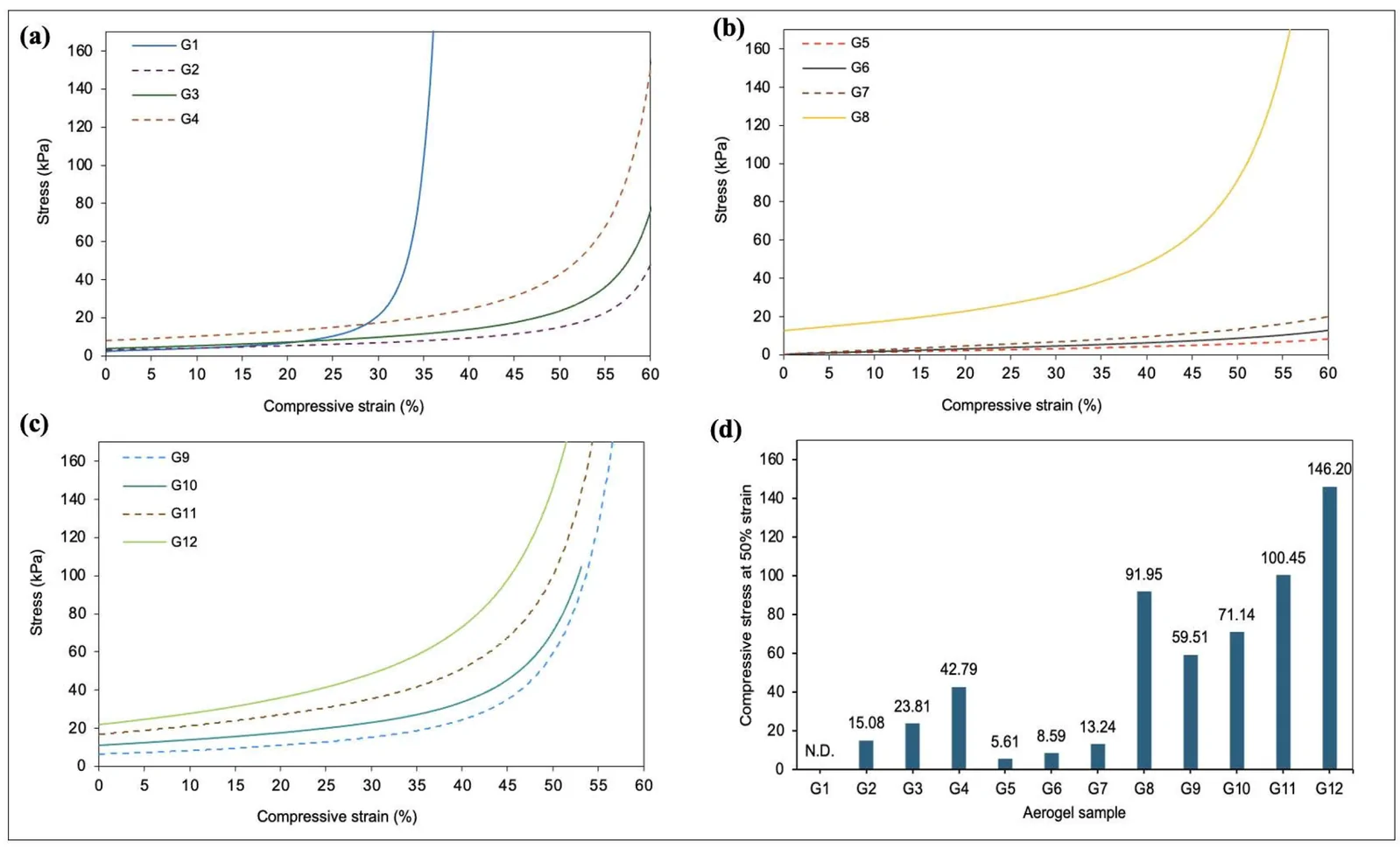
Compressive properties of GWF aerogels: compressive stress–strain curves of samples containing (**a**) 1.0 wt.% GWF (G1–G4), (**b**) 2.0 wt.% GWF (G5–G8), and (**c**) 3.0 wt.% GWF (G9–G12); and (**d**) compressive stress at 50% strain (σ_50_) for samples G2–G12. The PVA concentrations within each GWF series were 0.10, 0.25, 0.50, and 1.00 wt.%. N.D., not determined.

The uniaxial compressive stress–strain curves of the twelve GWF aerogels are presented in Figure 5a–c. The curves generally exhibited nonlinear, J-shaped profiles, characterized by relatively low compressive stresses at small strains followed by progressively steeper increases at larger strains. At the initial stage of compression, deformation was likely accommodated primarily by bending and rearrangement of the glass wool fibers, together with the deformation of the PVA-associated inter-fiber junctions. As compression proceeded, progressive structural densification and the formation of additional fiber–fiber contacts likely increased the load-bearing capacity of the fibrous framework. The pronounced increase in stress at larger strains was therefore attributed mainly to structural densification rather than to a distinct failure event. Similar composition-dependent compressive behavior has been reported for highly porous PVA-bound aerogels derived from recycled tire fibers [40].

Because neither a clearly defined linear elastic region nor a distinct failure point was consistently observed across the formulations, the compressive stress at 50% strain, σ_50_, was used as a common comparative indicator of resistance to compression and structural densification. The σ_50_ value of G1 was not determined because its stress–strain curve did not reach 50% strain. Among the remaining formulations, σ_50_ ranged from 5.61 kPa for G5 to 146.20 kPa for G12 (Figure 5d).

Among the samples containing 1.0 wt.% GWF, σ_50_ increased from 15.08 kPa for G2 to 23.81 kPa for G3 and 42.79 kPa for G4 as the PVA concentration increased from 0.25 to 1.00 wt.% (Figure 5a,d). Within this composition range, increasing the PVA concentration was associated with greater resistance of the relatively sparse fibrous network to compression. This trend may reflect an increased contribution from PVA-mediated inter-fiber interactions, which could enlarge the effective contact area, restrict relative fiber displacement, and facilitate load transfer through the porous network [47].

At 2.0 wt.% GWF, σ_50_ increased gradually from 5.61 kPa for G5 to 8.59 and 13.24 kPa for G6 and G7, respectively, as the PVA concentration increased from 0.10 to 0.50 wt.%. A pronounced increase to 91.95 kPa was observed for G8 containing 1.00 wt.% PVA, suggesting that the contribution of PVA to network reinforcement became substantially more pronounced at the highest PVA concentration investigated (Figure 5b,d).

At 3.0 wt.% GWF, σ_50_ increased systematically from 59.51 kPa for G9 to 71.14, 100.45, and 146.20 kPa for G10, G11, and G12, respectively, as the PVA concentration increased from 0.10 to 1.00 wt.% (Figure 5c,d). This systematic increase may reflect a progressively greater contribution of PVA to inter-fiber cohesion and network reinforcement within the denser 3.0 wt.% GWF formulation. The localized film-like and bridge-like features observed on the fiber surfaces and at fiber–fiber contact regions in the representative SEM images provide qualitative support for the proposed role of PVA in improving network cohesion (Figure 1b).

The effect of GWF content on σ_50_ depended on the PVA concentration. At a fixed PVA concentration of 1.00 wt.%, σ_50_ increased from 42.79 kPa for G4 to 91.95 kPa for G8 and 146.20 kPa for G12 as the GWF content increased from 1.0 to 3.0 wt.% (Figure 5d). This trend is consistent with the corresponding increase in bulk density and decrease in calculated porosity reported in Table 1. A higher fiber content may have increased the number of load-bearing fibers and fiber–fiber contacts per unit volume, thereby facilitating load transfer during compression [47].

However, a monotonic dependence on GWF content was not observed at PVA concentrations of 0.25 and 0.50 wt.%. At 0.25 wt.% PVA, σ_50_ decreased from 15.08 kPa for G2 to 8.59 kPa for G6 before increasing to 71.14 kPa for G10. Similarly, at 0.50 wt.% PVA, σ_50_ decreased from 23.81 kPa for G3 to 13.24 kPa for G7 and subsequently increased to 100.45 kPa for G11. These nonmonotonic responses suggest that the compressive behavior cannot be explained by GWF content or bulk density alone but likely reflects the combined effects of GWF content, PVA concentration, inter-fiber contact architecture, and local structural heterogeneity.

Overall, the compressive resistance of the GWF aerogels was governed by the combined effects of composition and the resulting fibrous network structure rather than by either GWF content or PVA concentration alone. The results further suggest a composition-dependent trade-off between structural reinforcement and the openness of the inter-fiber structure observed by SEM, which is relevant to the crude-oil absorption and cyclic-reuse performance discussed in the following section. Because the liquid-accessible pore volume was not directly measured, a quantitative relationship between pore accessibility and compressive behavior cannot be established from the present data.

It should be emphasized that the present uniaxial compression tests characterize the initial monotonic compressive response of the GWF aerogels rather than their long-term mechanical durability. Accordingly, the reported σ_50_ values are used as comparative indicators of compressive resistance and are not interpreted as measures of resilience, strain recovery, energy dissipation, stress retention, or fatigue resistance under repeated loading. Recent studies on mechanically engineered cellular structures and fiber-containing composites have highlighted the importance of structural organization, deformation behavior, and energy dissipation when evaluating mechanical performance beyond a single loading event [20,22]. In the present study, application-relevant repeated deformation was assessed separately through the absorption–mechanical squeezing experiments described in Section 2.3.2, which provide complementary information on structural integrity during repeated oil-recovery operations. These squeezing experiments, however, are distinct from controlled cyclic compression tests and are therefore not used here to infer intrinsic resilience or long-term fatigue behavior. Further cyclic loading–unloading studies would provide additional insight into the long-term mechanical durability of the GWF aerogels.

### 2.3. Oil Absorption Performance of Glass Wool Fiber Aerogels

#### 2.3.1. Crude-Oil Absorption Capacity

**Figure 6:**
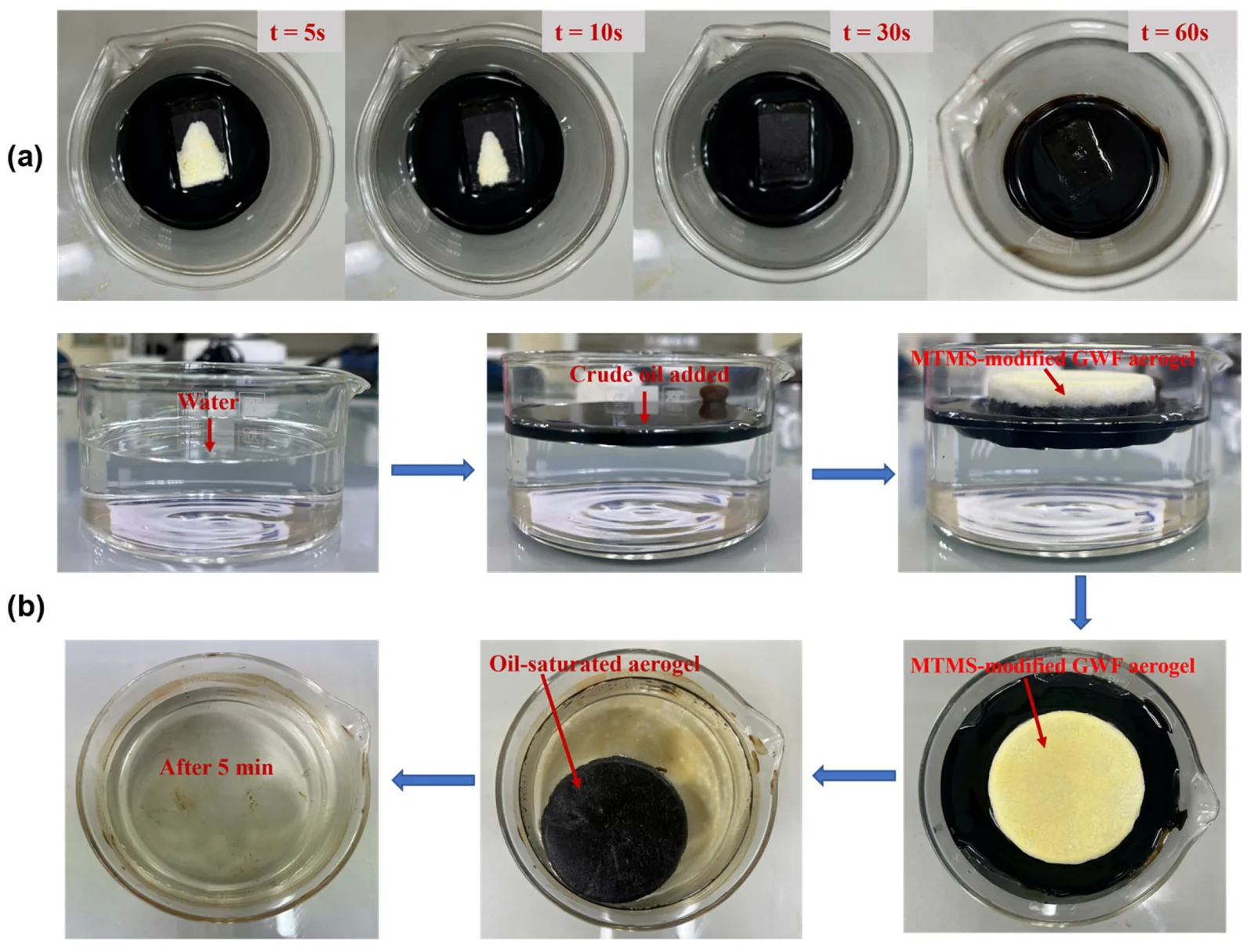
Visual demonstration of rapid crude-oil uptake and floating oil removal from water by an MTMS-modified GWF aerogel. (**a**) Photographic sequence showing rapid crude-oil uptake during the first 60 s. (**b**) Qualitative demonstration of floating crude-oil removal from water using the MTMS-modified GWF aerogel.

A representative MTMS-modified GWF aerogel progressively changed from its initial pale-yellow appearance to dark brown and subsequently black during the first 60 s of contact with VSP crude oil (Thang Long Light) (Figure 6a). The progressive darkening of the visible aerogel surface is consistent with rapid initial oil wetting and penetration into the fibrous aerogel.

The maximum oil absorption capacity, Q_max_, varied from 18.86 ± 1.50 to 55.12 ± 1.57 g/g across the twelve formulations (Table 1). G2, containing 1.0 wt.% GWF and 0.25 wt.% PVA, exhibited the highest capacity, whereas G12, containing 3.0 wt.% GWF and 1.0 wt.% PVA, showed the lowest value. These results demonstrate that the oil absorption performance was strongly dependent on the relative GWF and PVA contents.

At each PVA concentration, Q_max_ decreased with increasing GWF content. For example, at 0.25 wt.% PVA, the capacity decreased from 55.12 ± 1.57 g/g for G2 to 34.58 ± 1.80 g/g for G6 and 22.36 ± 2.36 g/g for G10 as the GWF content increased from 1.0 to 3.0 wt.%. Similarly, at 1.0 wt.% PVA, Q_max_ decreased from 43.64 ± 3.47 g/g for G4 to 28.01 ± 1.02 g/g for G8 and 18.86 ± 1.50 g/g for G12. This systematic reduction in oil uptake coincided with the corresponding increase in bulk density and decrease in calculated porosity reported in Table 1. Increasing the GWF loading increased the solid mass fraction per unit volume and was associated with a lower gravimetric oil-storage capacity. Because oil-accessible pore volume was not measured directly, this trend is interpreted as a qualitative composition–structure–performance relationship rather than as evidence of a quantified change in accessible pore volume. Comparable relationships between solid loading, density, porosity, and oil uptake have been reported for other fiber-based aerogels [17,48].

In contrast, the effect of PVA concentration depended on the GWF content, and no common monotonic trend was observed across the three GWF series. At 2.0 wt.% GWF, Q_max_ initially increased from 31.29 ± 1.58 to 34.58 ± 1.80 g/g as the PVA concentration increased from 0.1 to 0.25 wt.%, but subsequently decreased to 32.34 ± 1.46 and 28.01 ± 1.02 g/g at 0.5 and 1.0 wt.% PVA, respectively. At 3.0 wt.% GWF, the absorption capacity decreased progressively with increasing PVA content. These variations suggest competing effects between binder-mediated structural reinforcement and composition-dependent changes in the inter-fiber void geometry. A moderate PVA concentration may strengthen inter-fiber junctions while retaining relatively open inter-fiber spaces, whereas higher-PVA contents may promote the formation of more extensive film-like and bridge-like regions on the fiber surfaces and at fiber junctions, as observed in Figure 1b. These binder-rich regions may enhance structural cohesion while locally narrowing some of the inter-fiber openings visible in the SEM images. Because three-dimensional pore volume and pore connectivity were not directly quantified, this interpretation remains qualitative. Thus, the optimum absorption performance did not correspond simply to the lowest or highest PVA content, but rather appeared to reflect a balance between maintaining an open fibrous morphology and sufficient structural integrity.

Two-way ANOVA confirmed significant effects of GWF content ($F\left(224\right)=280.44,\;p<0.001$), PVA content ($F\left(324\right)=24.77,\;p<0.001$), and their interaction ($F\left(624\right)=13.49,\;p<0.001$) on $Q_{max}$. The significant GWF × PVA interaction confirms that the effect of PVA on $Q_{max}$ depended on GWF content, consistent with the nonmonotonic trends observed across the formulation matrix.

The relatively high gravimetric absorption capacities of the MTMS-modified GWF aerogels are consistent with their low bulk density, high calculated porosity, and open inter-fiber morphology observed by SEM. The visible inter-fiber spaces may provide pathways for oil ingress and physical retention within the fibrous framework, while MTMS-derived surface modification imparted strong water repellency, as indicated by the measured water contact angles. However, because pore-size distribution, three-dimensional pore connectivity, and oil-accessible pore volume were not directly measured, the contribution of the pore network to liquid transport and storage can only be interpreted qualitatively rather than as a quantitatively established transport mechanism. Similar relationships among high calculated porosity, open fibrous morphology, surface modification, and oil uptake have been reported for other aerogel-based sorbents [17,48,49].

**Figure 7:**
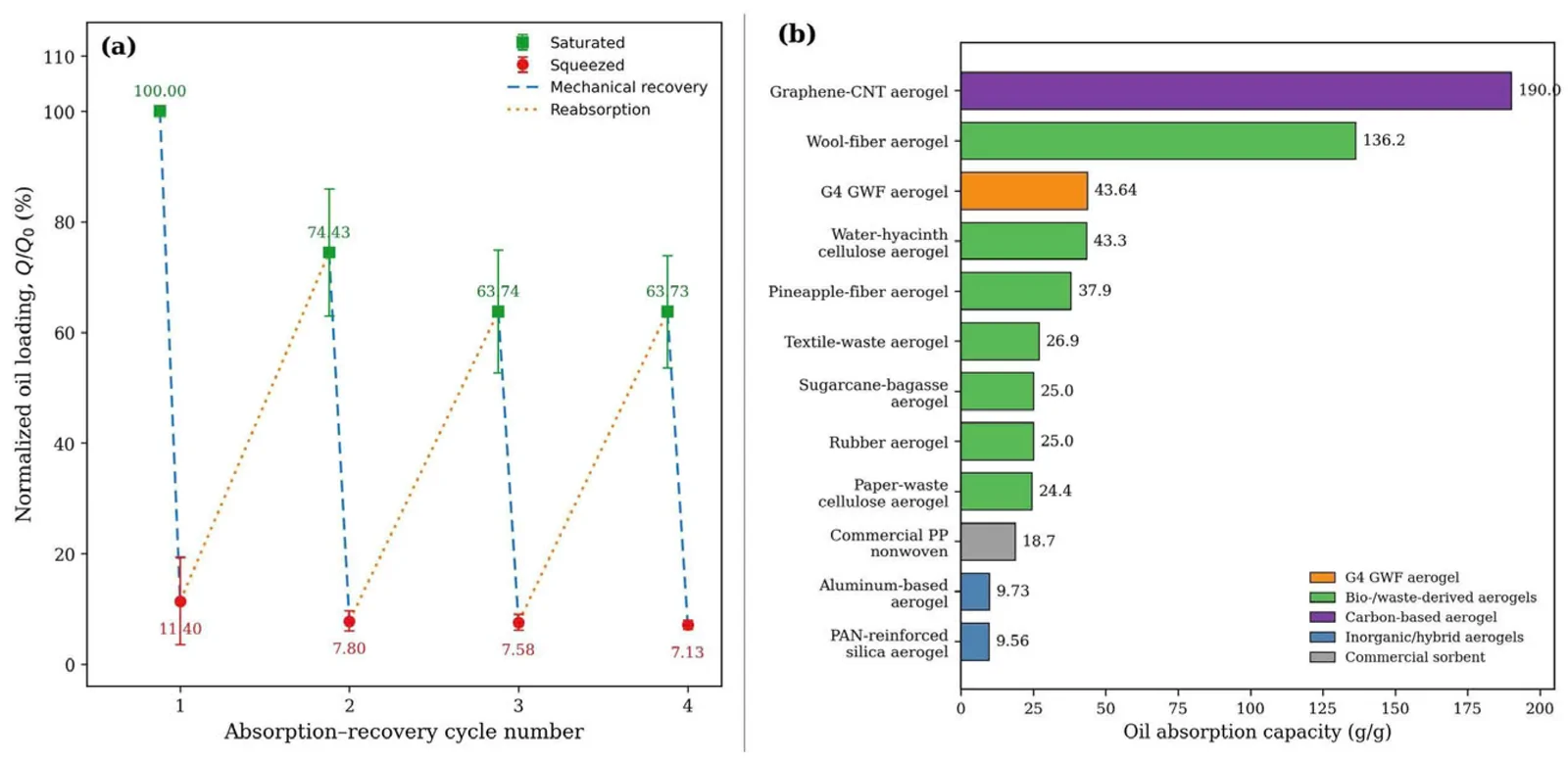
Oil absorption and cyclic reusability of the MTMS-modified GWF aerogel. (**a**) Normalized oil loading of G4 during four absorption–mechanical squeezing cycles. (**b**) Contextual comparison of the gravimetric oil absorption capacity of G4 with reported sorbents. The literature values in panel (**b**) were obtained using different sorbates, specimen preparation procedures, sample characteristics, and testing protocols and are therefore not directly comparable.

For literature benchmarking, G4 was selected as a representative formulation because it combined a relatively high first-cycle absorption capacity with sufficient structural integrity to complete four absorption–recovery cycles. As shown in Figure 7b, its VSP crude-oil absorption capacity of 43.64 ± 3.47 g/g falls within the broad range reported for porous oil sorbents. Reported values include 43.3 g/g for a water-hyacinth cellulose aerogel [48], 37.9 g/g for a pineapple-fiber aerogel [50], 26.9 g/g for a textile-waste-fiber aerogel [32], approximately 25.0 g/g for sugarcane-bagasse and waste-tire-fiber aerogels [17,40], 24.4 g/g for a paper-waste cellulose aerogel [49], 18.7 g/g for a commercial nonwoven polypropylene sorbent [51], 9.73 g/g for an aluminum-based aerogel [52], and 9.56 g/g for a PAN-reinforced silica aerogel [53]. Higher capacities of 136.2 and approximately 190 g/g have been reported for wool fiber and graphene-CNT aerogels, respectively [36,54]. Importantly, these literature values were obtained using different oils or organic liquids, specimen compositions and preparation procedures, sample densities and geometries, immersion and drainage conditions, and capacity-determination protocols. Therefore, Figure 7b is intended only as a contextual literature benchmark and should not be interpreted as a direct performance ranking. Under the testing conditions used in the present study, G4 combined a first-cycle capacity of 43.64 ± 3.47 g/g with sufficient structural integrity for repeated mechanical oil recovery.

#### 2.3.2. Oil-Recovery Efficiency and Reusability

Cyclic absorption–mechanical squeezing tests provided an application-specific assessment of structural integrity during repeated oil-recovery operations and revealed a clear trade-off between initial gravimetric oil absorption capacity and resistance to repeated squeezing. G1 and G2, which contained the lowest overall solid contents and exhibited high first-cycle capacities, lost their structural integrity during the first mechanical squeezing step. In contrast, G3–G12 retained sufficient integrity to complete four consecutive absorption–recovery cycles, although none could be reused for an additional cycle after the fourth recovery step. These results suggest that the low-solid formulations, which exhibited lower bulk densities and higher calculated porosities, were associated with high initial gravimetric oil uptake but provided insufficient structural reinforcement to withstand repeated mechanical squeezing during oil recovery. The complete cycle-by-cycle incremental oil uptake capacities and oil-recovery efficiencies for all formulations are presented in Table 2.

Among the formulations that completed four cycles, G4 exhibited the highest mean fourth-cycle incremental oil uptake, reaching 24.72 ± 6.38 g/g. Together with its relatively high first-cycle capacity of 43.64 ± 3.47 g/g, this result indicates a favorable balance between gravimetric oil uptake and inter-fiber network reinforcement. G4 was therefore selected as the representative formulation for the detailed cyclic oil-loading analysis shown in Figure 7a.

Figure 7a presents the total oil loading of G4 normalized to its first saturated state. For graphical reference, the initial dry and first saturated states were plotted at step 0 as 0% and 100%, respectively. Following the first mechanical squeezing step, the normalized residual oil loading decreased to 11.40%, while subsequent reabsorption restored the loading to 74.43%. The re-saturated loadings after the next two recovery steps were 63.74% and 63.73%, whereas the corresponding residual loadings after squeezing were 7.80% and 7.58%. After the fourth squeezing step, the residual loading decreased to 7.13%. No subsequent re-saturated state was obtained because the specimen no longer retained sufficient structural integrity for an additional absorption–recovery cycle.

The largest reduction in re-saturated oil loading occurred after the first mechanical squeezing step, indicating that the initial compression caused substantial and partly irreversible densification of the porous fibrous structure. This reduction is consistent with a possible decrease in oil-accessible void space, while residual oil retained within the compressed structure may also have reduced the capacity for subsequent uptake. Similar reductions in cyclic uptake have been attributed to compression-induced structural collapse and incomplete oil removal in textile-waste-fiber [32] and paper-waste cellulose aerogels [49]. Because the post-compression pore structure was not quantitatively characterized, the relative contributions of structural densification and residual oil retention cannot be distinguished from the present data.

After the initial reduction, the very small difference between the subsequent re-saturated loadings of 63.74% and 63.73% suggests that the aerogel approached a comparatively stable cyclic uptake state. These similar values indicate the stabilization of the gravimetric oil-loading behavior during the later cycles rather than demonstrating that the accessible pore volume itself remained constant.

Despite the reduction in re-saturated oil loading, G4 maintained oil-recovery efficiencies of 88.13–89.61% over the four cycles (Table 2), indicating that mechanical squeezing removed most of the oil absorbed in each cycle. However, the lower re-saturated loadings after the first cycle show that effective oil recovery did not restore the original oil-loading capacity. This cyclic behavior therefore reflects a trade-off between gravimetric oil uptake and structural reinforcement: low-solid formulations were associated with high initial oil uptake, whereas greater structural reinforcement improved resistance to repeated mechanical squeezing.

Taken together, the results reveal a composition-dependent trade-off among gravimetric oil uptake, compressive resistance, and cyclic reusability. At the low-solid end of the composition range, high oil uptake was accompanied by insufficient structural durability, whereas G12 exhibited the highest compressive resistance (σ_50_ = 146.20 kPa) but the lowest Q_max_ (18.86 ± 1.50 g/g), highlighting the trade-off between structural resistance and gravimetric oil uptake. In comparison, G4 provided a more favorable balance among these competing properties, combining a relatively high Q_max_ of 43.64 ± 3.47 g/g with sufficient structural integrity to complete four absorption–recovery cycles and the highest fourth-cycle incremental uptake (ΔQ_4_ = 24.72 ± 6.38 g/g) among the reusable formulations. These results indicate that maximizing either initial gravimetric oil uptake or mechanical reinforcement alone is insufficient; rather, effective cyclic oil absorption requires a balance between oil-storage performance and inter-fiber network stability. Nevertheless, the inability of the reusable formulations to withstand an additional cycle beyond the fourth recovery step indicates limited application-specific reusability under repeated squeezing. Further improvement should therefore focus on strengthening inter-fiber connections while retaining the open fibrous morphology associated with high gravimetric oil uptake.

Beyond the cyclic recovery tests, Figure 6b provides a qualitative demonstration of floating crude-oil removal from water using the selected MTMS-modified GWF aerogel. The aerogel remained afloat at the oil–water interface and absorbed the floating oil, resulting in a marked reduction in the visible oil layer after 5 min. This behavior is consistent with its low density, porous structure, and hydrophobic–oleophilic surface characteristics, which promote buoyancy and preferential oil wetting. Figure 6b therefore supports the potential application of the aerogel for removing floating oil from water; however, it should be regarded as a proof-of-concept demonstration rather than a quantitative assessment of oil-removal efficiency.

#### 2.3.3. Oil Absorption Kinetics

**Figure 8:**
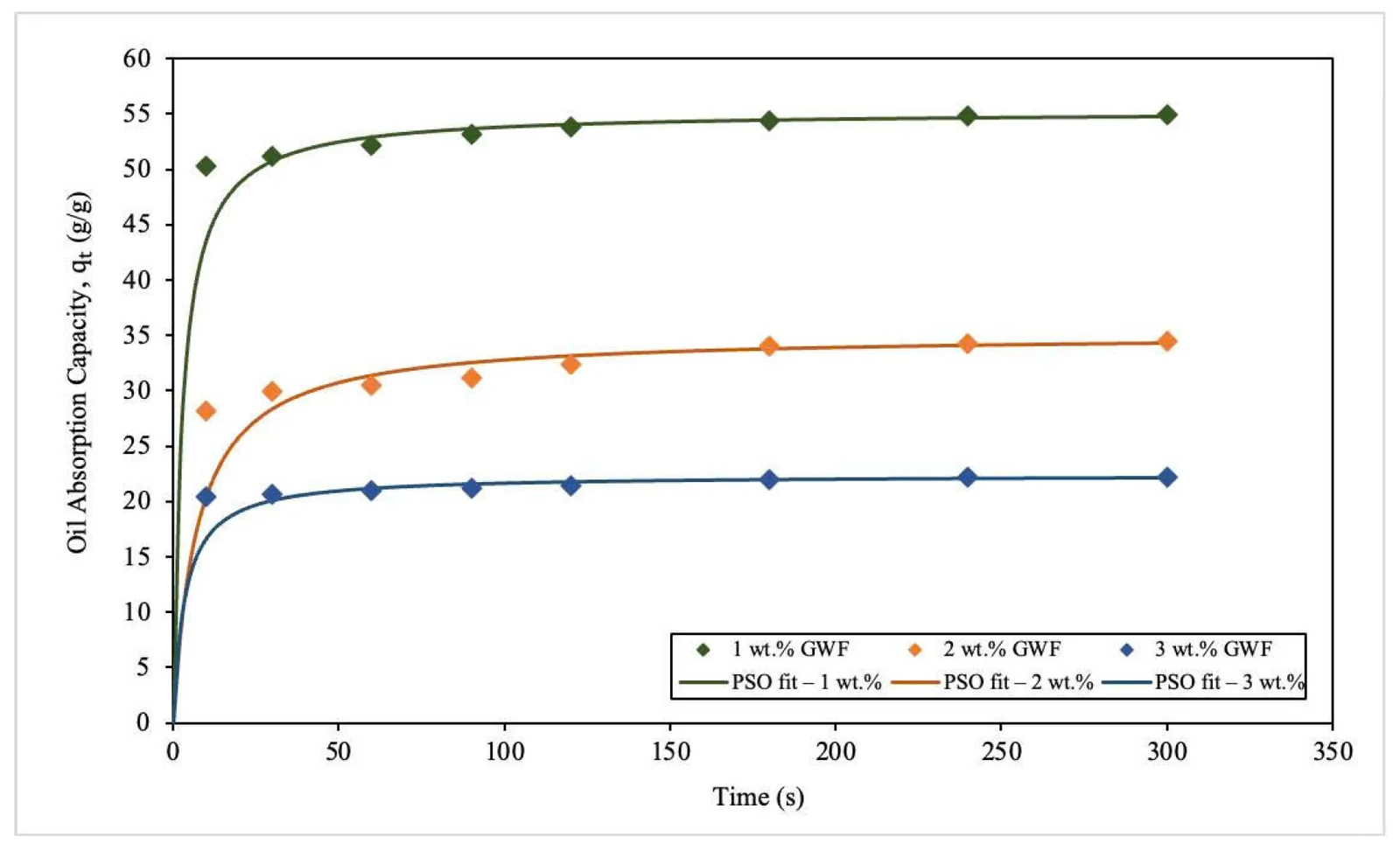
Time-dependent crude-oil absorption capacities of MTMS-modified GWF aerogels with varying GWF contents at a fixed PVA content of 0.25 wt.%.

The time-dependent crude-oil absorption capacities of samples G2, G6, and G10, containing 1.0, 2.0, and 3.0 wt.% GWF, respectively, at a fixed PVA concentration of 0.25 wt.%, are presented in Figure 8. All three samples exhibited rapid oil uptake during the initial stage, reaching approximately 81–91% of their experimental equilibrium capacities within the first 10 s. Thereafter, the uptake rates decreased progressively, and the absorption capacities gradually approached plateau values. The experimental equilibrium absorption capacities, Q_e,exp_, were 54.89 ± 1.90, 34.51 ± 2.90, and 22.23 ± 0.68 g/g for G2, G6, and G10, respectively (Table 3). Thus, the equilibrium absorption capacity decreased substantially as the GWF content increased from 1.0 to 3.0 wt.% at the fixed PVA concentration.

At 300 s, crude-oil uptake differed significantly among G2, G6, and G10 ($F\left(26\right)=97.98,\;p<0.001$), with Tukey’s honestly significant difference (HSD) test confirming significant pairwise differences among the three formulations.

The rapid initial uptake is consistent with fast oil wetting and liquid entry into the open inter-fiber spaces of the aerogel network. Capillary-assisted transport may also contribute to rapid liquid uptake in fibrous porous sorbents [14]. However, dynamic liquid–surface behavior can depend strongly on interfacial and operating conditions, as illustrated by a recent droplet-dynamics study on superhydrophobic surfaces [16]. Because advancing and receding contact angles, oil-contact-angle measurements, and time-resolved capillary-uptake experiments were not performed in the present study, capillary transport is therefore treated as a possible contribution to liquid uptake rather than as a quantitatively established transport pathway. As absorption progressed, the uptake rate decreased toward a plateau, consistent with progressive liquid filling and retention within available void spaces and increasing resistance to further transport. The decrease in $Q_{e,exp}$ with increasing GWF content coincided with the corresponding increase in bulk density and decrease in calculated porosity reported in Table 1. However, because pore-size distribution, three-dimensional pore connectivity, and oil-accessible pore volume were not independently measured, these trends are interpreted as qualitative composition–structure–performance relationships rather than as direct evidence of a quantitatively resolved pore-transport mechanism.

**Figure 9:**
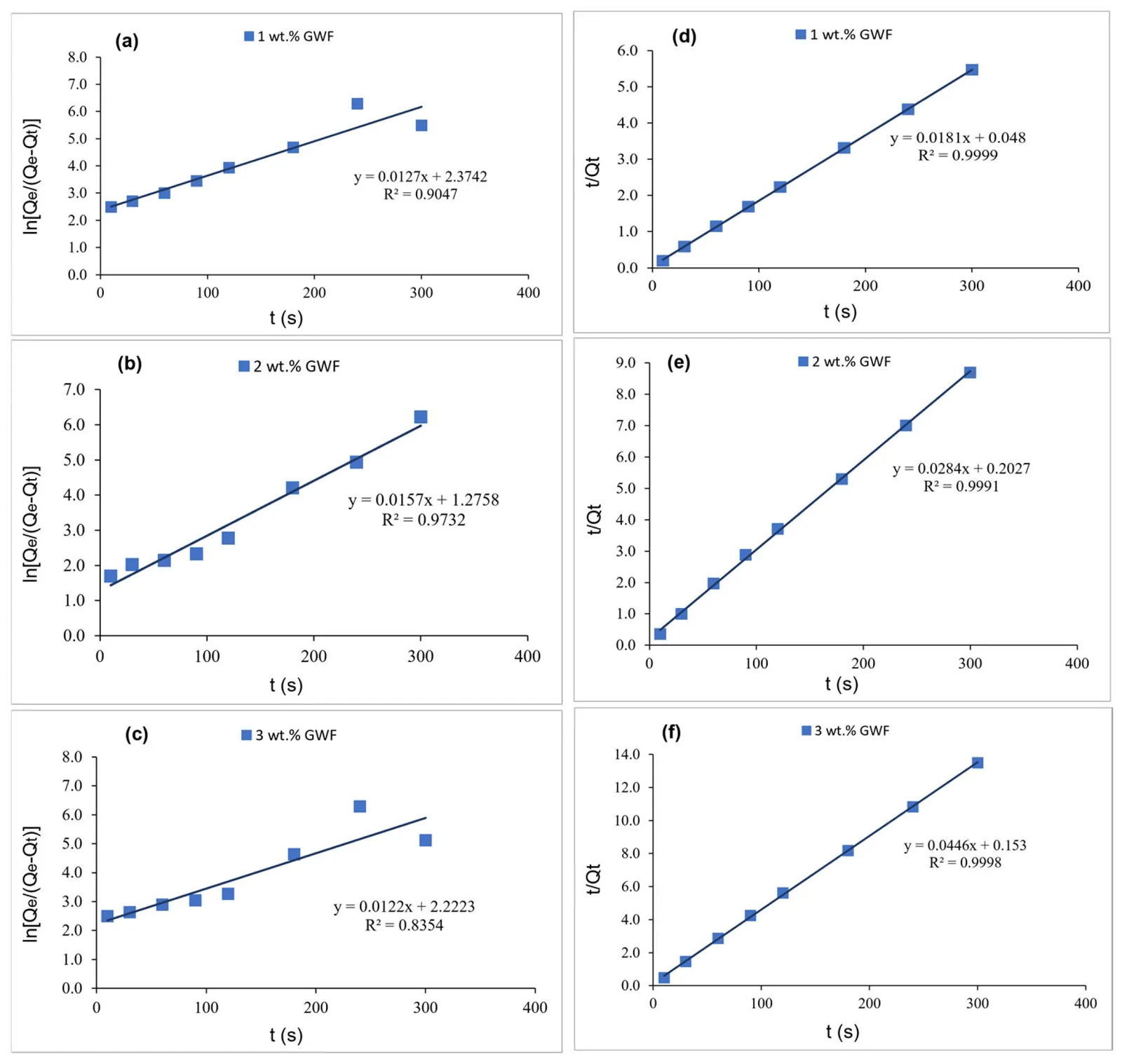
Linearized kinetic plots for crude-oil absorption by samples G2, G6, and G10: (**a**–**c**) pseudo-first-order model and (**d**–**f**) pseudo-second-order model.

The conventional linearized PFO and PSO analyses yielded the parameters summarized in Table 3 and the corresponding plots shown in Figure 9. The linearized PSO model gave higher $R^{2}$ values than the PFO model and calculated equilibrium capacities close to the experimental values. Because kinetic-model linearization can affect the apparent goodness of fit [55,56], the experimental uptake data were additionally reanalyzed by direct nonlinear regression. For G2, G6, and G10, the nonlinear PSO model yielded $R^{2}$ values of 0.7191, 0.6858, and 0.5784, respectively, compared to 0.4290, 0.4201, and 0.3132 for the nonlinear PFO model. The nonlinear PSO fits also produced consistently lower RMSE and SSE values (Table 3), indicating a better empirical description of the measured uptake data among the two models evaluated. Similar trends, with PSO providing a better empirical fit than PFO, have been reported for hydrophobically modified fibrous aerogels derived from waste-tire fibers and harakeke nanocellulose [40,57].

Importantly, the better empirical performance of the PSO model is not interpreted as evidence that chemisorption controls crude-oil uptake. Kinetic-model parameters and goodness-of-fit statistics alone are insufficient to establish a unique transport or molecular mechanism, particularly when linearized representations are considered [55,56]. More broadly, a recent study of sorption in heterogeneous porous media showed that uptake behavior can be influenced by coupled effects of pore characteristics and sorbate–surface interactions [58], further highlighting the need for caution when assigning a unique mechanism from empirical kinetic fits. For the present GWF aerogels, the observed behavior is instead consistent with physical wetting, liquid infiltration into inter-fiber voids, and retention within the fibrous framework [14,15].

**Figure 10:**
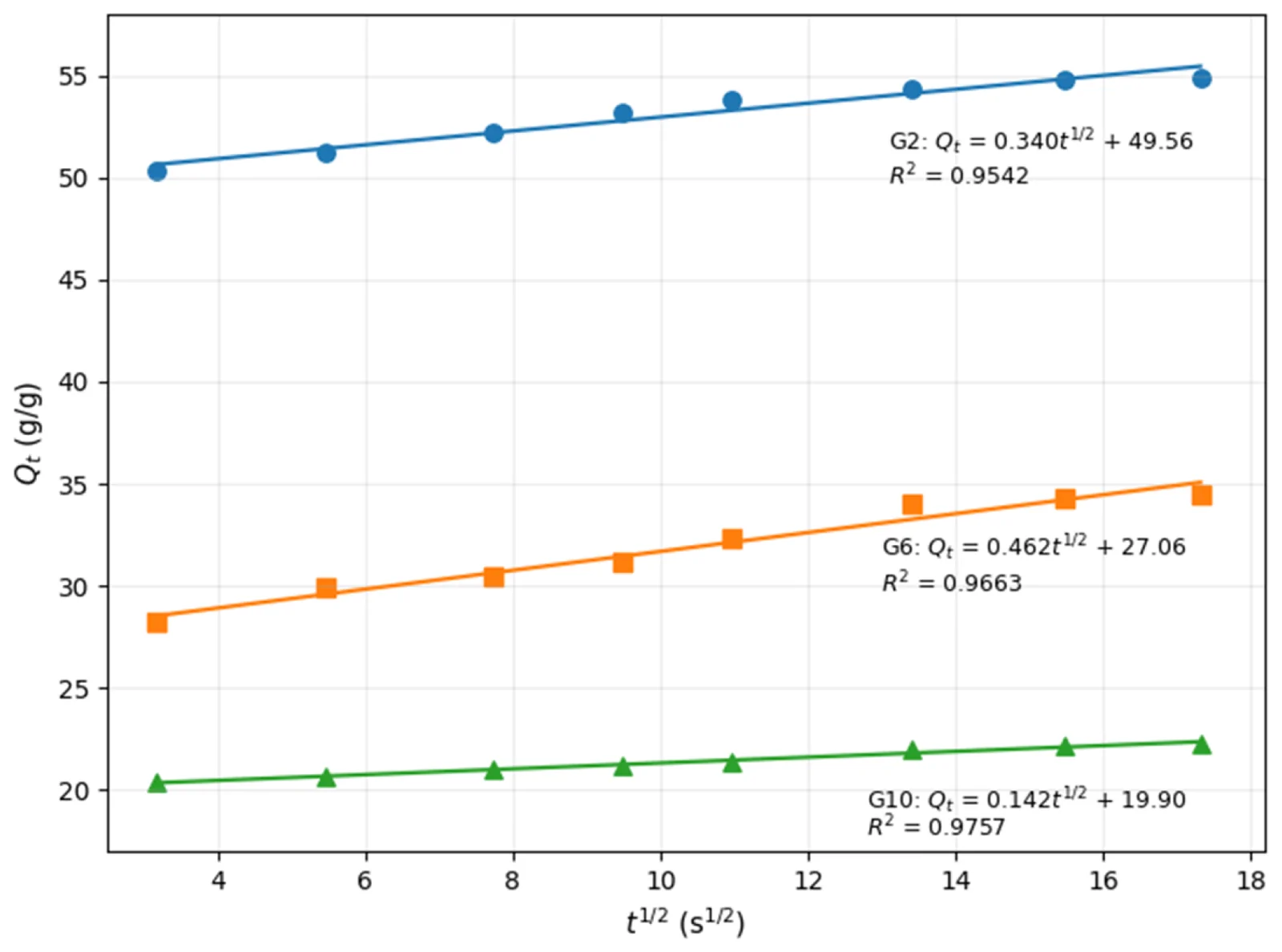
Weber–Morris-type square-root-of-time plots for crude-oil uptake by MTMS-modified GWF aerogels G2, G6, and G10 over the measured interval of 10–300 s.

To provide an additional transport-oriented assessment, a Weber–Morris-type square-root-of-time analysis [59] was performed by plotting $Q_{t}$ against $t^{1/2}$ over the measured interval of 10–300 s (Figure 10). The fitted $R^{2}$ values were 0.9542, 0.9663, and 0.9757 for G2, G6, and G10, respectively, with apparent $k_{id}$ values of 0.340, 0.462, and 0.142 (g/g)·$s^{-1/2}$. The corresponding intercepts, C, were 49.56, 27.06, and 19.90 g/g, respectively (Table 3). Although the square-root-of-time relationships were approximately linear over the measured interval, none of the fitted lines extrapolated through the origin. Therefore, the present data do not support assigning intraparticle diffusion as the sole rate-controlling process. Because the GWF aerogels are open fibrous monoliths rather than conventional particulate adsorbents, the Weber–Morris relation is interpreted here as an empirical transport diagnostic rather than as proof of a specific intraparticle-diffusion mechanism.

Taken together, the nonlinear kinetic fitting and Weber–Morris-type analysis indicate that no single fitted kinetic equation should be regarded as proof of a unique physicochemical uptake mechanism. The rapid initial uptake is consistent with predominantly physical wetting and infiltration, with a possible contribution from capillary-assisted transport [14,15], followed by a slower approach to the plateau. Notably, 81.8–91.8% of the 300 s uptake had already occurred by the first measurement at 10 s; therefore, the earliest transport stage was not sufficiently time-resolved for rigorous quantitative Lucas–Washburn analysis. Likewise, an additional Elovich fit was not used for mechanistic assignment because it would provide another empirical description without independently resolving the underlying transport pathway in the present open fibrous monolith. Future kinetic studies should include measurements at times shorter than 10 s, together with complementary pore-network and wettability characterization, to enable more rigorous quantitative evaluation of capillary-flow mechanisms.

### 2.4. Thermal Conductivity of Glass Wool Fiber Aerogels

The GWF aerogels exhibited low thermal conductivities ranging from 32.1 ± 0.1 mW/m·K for G1 to 38.5 ± 0.1 mW/m·K for G7 (Table 1). In highly porous fibrous materials, the effective thermal conductivity reflects combined contributions from solid-phase conduction through the fibrous/polymeric framework, gas-phase conduction through the air-filled voids, and radiative heat transfer. The relative contributions of these pathways depend on density, pore morphology, fiber arrangement, inter-fiber contacts, network architecture, and temperature [18]. The low-solid fraction of the present GWF aerogels is expected to restrict continuous solid-phase heat-transfer pathways, while the low thermal conductivity of air, approximately 26 mW/m·K near room temperature [60], contributes to their low effective thermal conductivity. However, the individual solid, gaseous, and radiative contributions were not measured separately in the present study.

The influence of composition on thermal conductivity was not uniform across the formulation matrix. At a fixed PVA content of 0.10 wt.%, increasing the GWF content from 1.0 to 3.0 wt.% increased the thermal conductivity from 32.1 mW/m·K for G1 to 35.8 mW/m·K for G5 and 38.2 mW/m·K for G9. Over the same series, the bulk density increased from 0.014 to 0.036 g/cm^3^ and the calculated porosity decreased from 99.39% to 98.49%. The higher solid fraction at increased GWF contents may promote fiber–fiber contacts and more continuous solid-phase heat-transfer pathways, although the connectivity of these pathways was not quantitatively characterized. Nevertheless, this trend was not reproduced uniformly at the other PVA concentrations, indicating that GWF content alone does not determine the effective thermal conductivity.

The effect of PVA was likewise nonmonotonic. Within the 1.0 wt.% GWF series, the thermal conductivity remained within a relatively narrow range of 32.1–33.4 mW/m·K. For the 2.0 wt.% GWF series, it increased from 35.8 to 38.5 mW/m·K before slightly decreasing to 38.2 mW/m·K, whereas for the 3.0 wt.% GWF series, it decreased from 38.2 to approximately 33.2 mW/m·K and then remained nearly unchanged. These trends suggest competing effects of solid fraction, local fiber–fiber contacts, binder-rich regions, and void geometry rather than a simple dependence on bulk density or calculated porosity. Recent studies further illustrate complementary aspects of multifunctional thermal management. Su et al. [35] demonstrated that thermally responsive wettability gradients and pore structure can regulate coupled thermal and moisture transport, whereas Guo et al. [34] showed that thermal insulation can be integrated with high mechanical strength and other functional characteristics in multifunctional composites. Although these systems differ substantially from the present GWF/PVA aerogels, they provide broader context for interpreting the coupling among porous architecture, mechanical integrity, and thermal performance.

Because pore-size distribution and three-dimensional pore connectivity were not quantitatively measured, the contributions of pore-size effects, gas-phase conduction, radiative transfer, and network connectivity cannot be quantitatively separated. The thermal behavior is therefore interpreted primarily in relation to the measured bulk density, calculated porosity, composition, and SEM-observed fibrous morphology rather than being assigned to a single heat-transfer mechanism.

**Figure 11:**
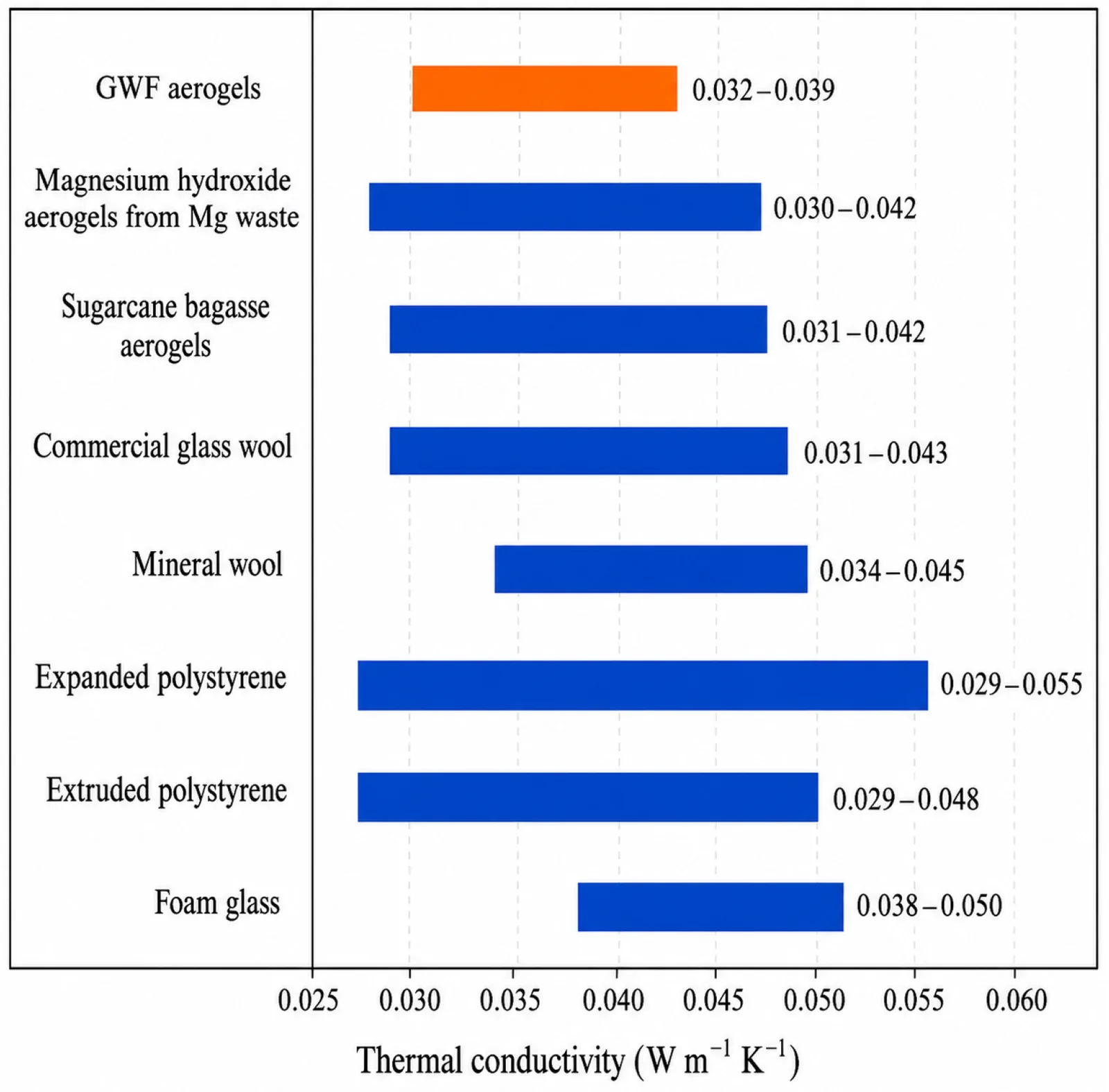
Thermal conductivity ranges of GWF aerogels compared to selected aerogels and commercial insulation materials.

Figure 11 compares the thermal-conductivity range of the prepared GWF aerogels with reported aerogels and conventional insulation materials. The measured range of 0.032–0.039 W/m·K was comparable to those reported for sugarcane-bagasse aerogels (0.031–0.042 W/m·K) [17] and magnesium-hydroxide aerogels recycled from magnesium waste (approximately 0.030–0.042 W/m·K) [61]. It also overlapped typical thermal-conductivity ranges reported for conventional fibrous and polymeric insulation materials, including mineral/glass wool and polystyrene-based insulation [62]. These direct thermal-conductivity comparisons are intended only as contextual benchmarks because thermal conductivity depends on measurement method, specimen density and geometry, temperature, moisture content, and other testing conditions. Beyond these direct thermal benchmarks, vertically aligned CNT architectures have been shown to influence electrochemical device performance [63], while the compositional modification of lithium solid electrolytes strongly affects ionic transport [64]. Overall, the measured values indicate that the GWF/PVA aerogels retained low thermal conductivity after PVA incorporation, freeze-drying, and MTMS modification, supporting the preservation of thermal-insulation functionality in the modified glass wool-based framework.

### 2.5. Study Limitations and Scale-Up Perspectives

Several aspects relevant to a more complete characterization and practical deployment remain to be established. Quantitative pore-network characterization, including specific surface area, pore-size and pore-volume distributions, and three-dimensional pore-network connectivity, was not performed in the present study. Accordingly, the relationships among pore architecture, mechanical response, oil uptake, and thermal transport are interpreted qualitatively on the basis of bulk density, calculated porosity, and SEM-observed morphology rather than as quantitatively resolved structure–property relationships. In addition, thermal stability by thermogravimetric analysis (TGA), flame response, chemical resistance, and long-term environmental aging were not evaluated. Therefore, the multifunctional performance demonstrated here is limited to the experimentally measured oil absorption and thermal-insulation properties, while these structural, durability, and safety aspects remain priorities for future investigation.

Broader implementation of multifunctional porous materials also requires the consideration of scalable processing and network-level performance optimization. Recent cellulose-based ionogel studies have demonstrated complementary strategies, including scalable solvent-induced layer-by-layer fabrication [65] and molecular-network reconstruction to balance mechanical integrity with functional performance [66]. Although these material systems differ substantially from the present GWF/PVA aerogels, they provide useful broader examples of scalable-processing strategies and network-level optimization in multifunctional porous materials. The GWF used in the present study was commercially supplied rather than recovered from a waste stream; therefore, no waste-utilization or waste-valorization benefit is claimed here.

In addition, the present fabrication and performance evaluation were conducted at laboratory scale, and no techno-economic or life-cycle assessment was performed. Future scale-up should therefore evaluate the material and energy requirements associated with freeze-drying and vapor-phase MTMS treatment, larger-format production, field-relevant oil-recovery performance, and the economic and environmental feasibility of the process relative to conventional sorbent systems.

## 3. Conclusions

Lightweight hydrophobic MTMS-modified GWF/PVA aerogels were successfully fabricated while retaining the glass wool fibrous framework as the primary structural component. The results demonstrate that the objective of identifying composition-dependent trade-offs among oil uptake, mechanical resistance, cyclic reusability, and thermal insulation was achieved. Rather than maximizing a single property, effective performance required a balance between maintaining an open fibrous network for oil uptake and providing sufficient inter-fiber reinforcement for mechanical recovery and reuse. In this respect, G4 provided a favorable balance, combining a relatively high crude-oil absorption capacity of 43.64 ± 3.47 g/g with sufficient structural integrity to complete four absorption–recovery cycles while maintaining oil-recovery efficiencies of 88.13–89.61%. MTMS modification imparted pronounced water repellency, while the aerogels retained low thermal conductivity after fabrication and surface modification, supporting their potential for combined oil-remediation and thermal-insulation applications. These findings are also consistent with the broader development of multifunctional lightweight porous materials [21,34]. Nevertheless, the present structure–property interpretations remain limited by the absence of quantitative pore-network characterization. Further work should address long-term durability, thermal and flame stability, chemical and environmental resistance, dynamic wettability and transport characterization, and scalable processing under field-relevant conditions.

## 4. Materials and Methods

### 4.1. Materials

Glass wool fibers (GWFs) were supplied by Cat Tuong Thermal and Acoustic Insulation Material Manufacturing Joint Stock Company (Ho Chi Minh City, Vietnam). Poly(vinyl alcohol) (PVA-124, analytical reagent grade, Mw ≈ 105,000 g/mol, degree of hydrolysis ≥ 97.0%, viscosity 54–66 mPa·s) was purchased from Xilong Scientific Co., Ltd. (Guangzhou, China). Methyltrimethoxysilane (MTMS) was purchased from Sigma-Aldrich (St. Louis, MO, USA). All materials were used as received without further purification. Deionized (DI) water was used to prepare all aqueous solutions. VSP crude oil (Thang Long Light, Hanoi, Vietnam), with a viscosity of 9.41 cSt at 40 °C, was supplied by the Vietnam Petroleum Institute (Hanoi, Vietnam) and used in the oil absorption experiments.

### 4.2. Fabrication and Surface Modification of GWF Aerogels

**Figure 12:**
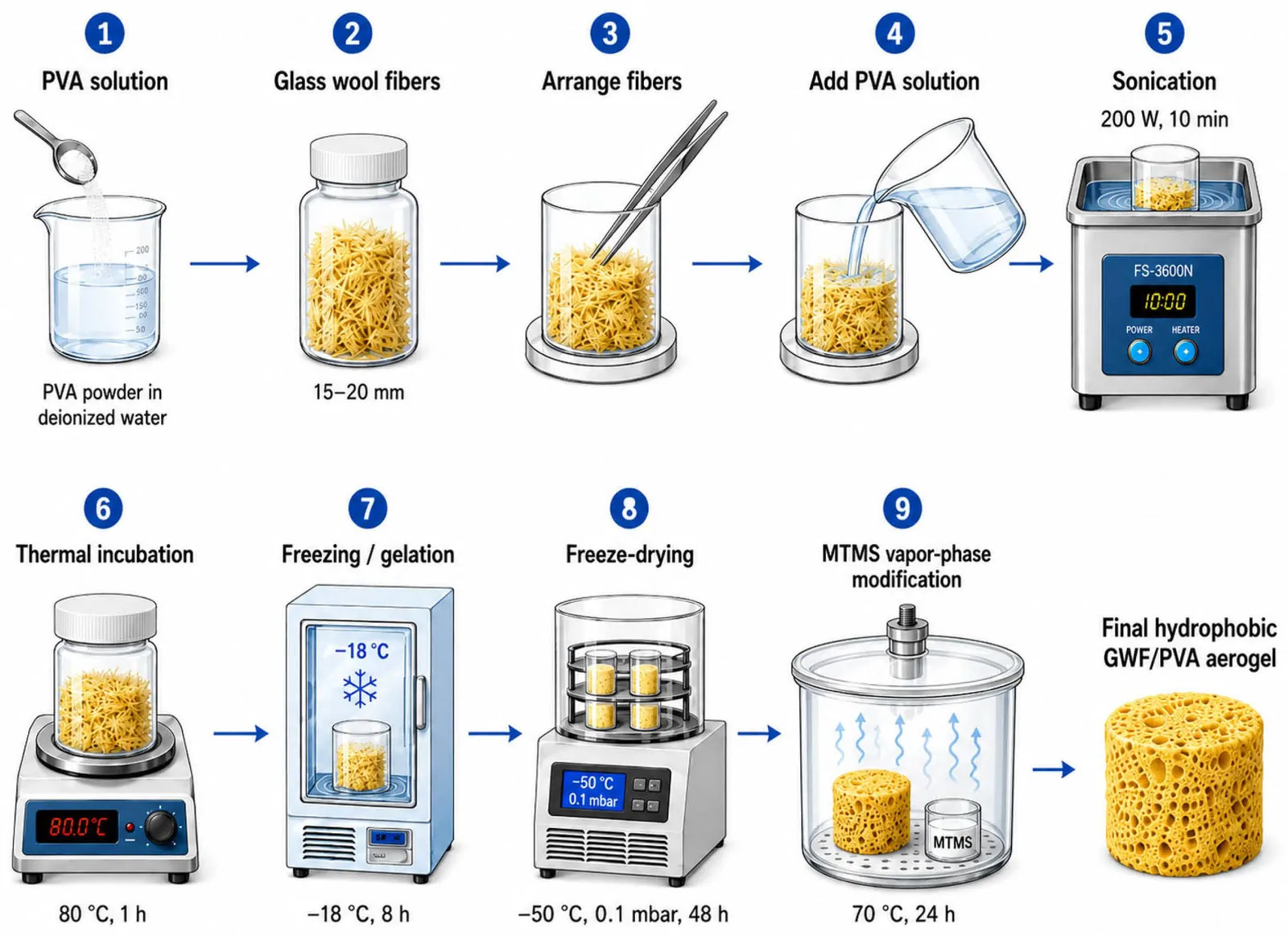
Schematic illustration of the fabrication and vapor-phase MTMS modification of hydrophobic GWF aerogels.

Figure 12 schematically illustrates the fabrication process of hydrophobic GWF aerogels. PVA powder was first dispersed in deionized water to obtain a homogeneous PVA solution. Glass wool mats were cut into fibers approximately 15–20 mm in length and placed in a mold. The fibers were then gently separated and manually dispersed to form a uniformly distributed fibrous network. Subsequently, the prepared PVA solution was poured over the glass wool fibers, ensuring thorough impregnation while minimizing the entrapment of air bubbles.

The suspension was sonicated using an FS-3600N ultrasonic processor (200 W; Shanghai Sonxi Ultrasonic Instruments Co., Ltd., Shanghai, China) for 10 min to ensure uniform dispersion of the glass wool fibers in the PVA solution, followed by thermal incubation at 80 °C for 1 h to facilitate hydrogen-bonding interactions between PVA hydroxyl groups and surface silanol (Si–OH) groups on the glass wool fibers.

The resulting suspension was frozen at −18 °C for 8 h to solidify the suspension and facilitate ice-template formation prior to freeze-drying. The frozen samples were subsequently freeze-dried at −50 °C and 0.1 mbar for 48 h to obtain lightweight porous aerogels. The GWF content and PVA concentration used in this study are summarized in Table 1.

The obtained aerogels were subsequently modified with MTMS via vapor-phase silanization to impart hydrophobicity. Each aerogel sample was placed in a sealed container together with an open glass vial containing MTMS. The sealed container was heated at 70 °C for 24 h to allow MTMS vapor to penetrate accessible regions of the porous network. In the presence of residual or adsorbed moisture, MTMS may undergo hydrolysis and subsequent condensation, introducing methyl-containing siloxane species at hydroxyl-containing regions of the GWF/PVA aerogel and thereby reducing the surface energy of the material [32,41,45,46]. Similar vapor-phase MTMS modification approaches have been reported for PVA-containing fibrous aerogels [32,41]. After treatment, the modified aerogels were removed from the container and used for subsequent characterization and oil absorption experiments.

### 4.3. Physical, Morphological, and Structural Characterization

Bulk density and calculated porosity were determined for the GWF/PVA aerogels prior to MTMS surface modification. The bulk density ($\rho_{bulk}$, g/cm^3^) of the aerogel was determined as the ratio of its measured dry mass to its geometric volume. The geometric volume of each cylindrical sample was calculated from its measured diameter and thickness. The porosity of the aerogel (Φ, %) was subsequently calculated using Equation (1) [67]:

(1) $$\Phi =\left(1-\frac{\rho_{bulk}}{\rho_{s}}\right)\times 100\%,$$

where $\rho_{bulk}$ is the bulk density of the dried aerogel and $\rho_{s}$ is the effective density of the GWF/PVA solid matrix.

The effective density of the solid matrix, $\rho_{s}$, was estimated using Equation (2), based on the true densities and weight percentages of the constituent materials:

(2) $$\rho_{s}\;=\frac{100}{\frac{C_{GWF}}{\rho_{GWF}}+\frac{C_{PVA}}{\rho_{PVA}}},$$

where $\rho_{GWF}\;$ = 2.5 g/cm^3^ and $\rho_{PVA}$ = 1.19 g/cm^3^ are the true densities of the glass wool fibers and PVA, respectively, while $C_{GWF}$ and $C_{PVA}$ are their corresponding weight percentages in the dried aerogel, with $C_{GWF}+\;C_{PVA}=100\%$.

The calculated porosity represents a density-derived estimate of the overall bulk void fraction and does not provide direct measurements of pore-size distribution, specific surface area, liquid-accessible pore volume, or three-dimensional pore-network connectivity. Quantitative structural characterization is therefore important for establishing rigorous structure–property relationships in porous materials [19,21].

The morphology and microstructure of the aerogel samples were characterized using Field-emission scanning electron microscopy (FE-SEM; TESCAN MIRA, Brno-Kohoutovice, Czech Republic). Prior to FE-SEM observation, the aerogel samples were cut into suitable sizes for imaging.

The crystal structure of the aerogel samples was characterized by X-ray diffraction (XRD) using a Malvern PANalytical diffractometer (Malvern Panalytical B.V., Almelo, The Netherlands) with Co Kα radiation (λ = 1.79 Å). The diffraction patterns were collected over a 2θ range of 5–80° with a step size of 0.02° and a scanning rate of 0.2° s^−1^.

Fourier-transform infrared (FTIR) spectra were recorded using an ALPHA II FTIR spectrometer (Bruker, Karlsruhe, Germany) over the wavenumber range of 4000–400 cm^−1^ to identify characteristic vibrational features associated with GWF, PVA, and MTMS-derived methyl-containing species in the aerogel samples.

Surface wettability was evaluated using an optical contact-angle goniometer (OCA 20, DataPhysics Instruments, Filderstadt, Germany). For the MTMS-modified GWF aerogels, a deionized-water droplet was gently deposited onto the sample surface, and the apparent WCA was determined from the droplet profile using the instrument software. At least five measurements were performed at different locations on each sample, the average value was reported. Pristine GWF and the unmodified GWF aerogel were also examined under the same water-droplet deposition procedure. For these highly wettable specimens, the droplets were rapidly imbibed into the porous fibrous structure before a stable droplet profile could be established; therefore, a stable apparent WCA was not reported.

### 4.4. Uniaxial Compression Testing

Uniaxial compression tests were performed using a universal testing machine (Instron 5500, Instron, Norwood, MA, USA) equipped with a 1 kN load cell. Before testing, the initial diameter and height of each cylindrical specimen were measured. The specimens were compressed at a crosshead speed of 1.0 mm/min, and the corresponding compressive stress–strain curves were recorded. Engineering compressive stress and strain were calculated from the measured force, initial cross-sectional area, and initial specimen height. The compressive stress at 50% strain (σ_50_) was used as a comparative parameter for specimens whose stress–strain curves reached 50% strain. The σ_50_ values were obtained from the stress–strain data at 50% strain, using linear interpolation where necessary. The compression tests in the present study were conducted under monotonic loading only; controlled loading–unloading cyclic compression tests were not performed.

### 4.5. Thermal Characterization

The thermal conductivity of the GWF aerogels was measured using a C-Therm TCi Thermal Conductivity Analyzer equipped with a T391 sensor (C-Therm Technologies Ltd., Fredericton, NB, Canada). Measurements were conducted under ambient conditions at 24.1–24.4 °C. For each measurement, the specimen was placed directly on the sensor head, with the test surface in contact with the sensing element, and stable contact was maintained throughout the test. Three consecutive measurements were performed for each formulation, and the results are reported as mean ± standard deviation in mW/m·K.

### 4.6. Oil Absorption Performance, Reusability, and Kinetic Analysis

The oil absorption performance of the MTMS-modified GWF aerogels was evaluated in terms of maximum oil absorption capacity, cyclic reusability, oil-recovery efficiency, absorption kinetics, and oil-removal performance. Cubic specimens measuring approximately 20 mm × 20 mm × 20 mm were weighed to determine their initial dry mass, $m_{0}$, and completely immersed in VSP crude oil (Thang Long Light) for 60 min under ambient conditions. The specimens were then removed using a stainless-steel mesh basket, allowed to drain in air for 30 s, and immediately weighed to determine their first-cycle saturated mass, $m_{s,1}$ [49]. The maximum oil absorption capacity, *Q*_max_, was calculated as

(3) $$Q_{max}=\frac{m_{s,1}-{\;m}_{0}}{m_{0}},$$

Cyclic reusability was evaluated through consecutive crude-oil absorption–mechanical squeezing cycles. In cycle n, the specimen was immersed in crude oil for 60 min, drained for 30 s, and weighed to obtain its saturated mass, $m_{s,n}$. The absorbed oil was subsequently recovered by mechanical squeezing, and the specimen was reweighed to determine its compressed-state mass, $m_{c,n}$. The compressed specimen was directly reused in the subsequent absorption cycle. Testing was discontinued when the specimen no longer retained sufficient structural integrity for further handling and reuse [49].

The incremental oil uptake during cycle n, Δ*Q*_n_, was calculated as

(4) $${\Delta Q}_{n}=\frac{m_{s,n}-{\;m}_{c,n-1}}{m_{0}}$$

where $m_{c,n-1}$ is the specimen mass after squeezing in the preceding cycle. For the first cycle, $m_{c,0}=m_{0}$, and therefore Δ*Q*_1_ = *Q*_max_.

The oil-recovery efficiency in cycle n, $\eta_{n}$, was calculated as

(5) $$\eta_{n}=\frac{m_{s,n}-m_{c,n}}{{m_{s,n}-m}_{0}}\times 100\%$$

To quantify the evolution of the total oil retained during cyclic absorption and mechanical squeezing, the saturated and compressed-state oil loadings were normalized to the first saturated state [68]:

(6) $$Q_{s,n}^{*}=\frac{m_{s,n}-m_{0}}{{m_{s,1}-m}_{0}}\times 100\%,$$

(7) $$Q_{c,n}^{*}=\frac{m_{c,n}-m_{0}}{{m_{s,1}-m}_{0}}\times 100\%,$$

All cyclic parameters were calculated separately for each replicate and are reported as mean ± standard deviation. Three specimens were tested unless structural failure reduced the number of valid replicates, in which case the actual sample number, n, was reported.

Oil absorption kinetics were investigated for samples G2, G6, and G10, containing 1.0, 2.0, and 3.0 wt.% GWF, respectively, at a fixed PVA content of 0.25 wt.%. Separate specimens with comparable dimensions and initial dry masses were used for each immersion time of 10, 30, 60, 90, 120, 180, 240, and 300 s. After immersion, each specimen was removed, allowed to drain in air for 30 s, and immediately weighed. The time-dependent oil absorption capacity, $Q_{t}$, was calculated as

(8) $$Q_{t}=\frac{m_{t}-m_{0}}{m_{0}},$$

where $m_{t}$ is the specimen mass after an immersion time t. The experimental data were fitted to the linearized PFO and PSO kinetic models:

(9) $${ln(Q}_{e}-Q_{t})=-k_{1}t+lnQ_{e},$$

(10) $$\frac{t}{Q_{t}}=\frac{1}{k_{2}.Q_{e}^{2}}+\frac{t}{Q_{e}},$$

where $Q_{e}\;$ is the equilibrium oil absorption capacity, and $k_{1}$ and $k_{2}$ are the pseudo-first-order and pseudo-second-order rate constants, respectively. The goodness of fit was assessed using the coefficient of determination ($R^{2}$) and the agreement between the model-calculated and experimentally determined equilibrium capacities.

The mean time-dependent uptake data were also reanalyzed using nonlinear PFO and PSO regression, with model performance assessed by the coefficient of determination ($R^{2}$), root-mean-square error (RMSE), sum of squared errors (SSE), and agreement between calculated and experimental equilibrium capacities.

To further examine the time dependence of liquid transport, the experimental uptake data were also evaluated using a Weber–Morris-type square-root-of-time relation:

(11) $$Q_{t}=k_{id}t^{1/2}+C$$

where $k_{id}$ is the apparent square-root-of-time slope parameter and C is the intercept obtained from the linear fit.

The oil-removal performance of the MTMS-modified GWF aerogel was evaluated using a model oil–water system. Briefly, 200 mL of deionized (DI) water and 20 mL of crude oil were placed in a 250 mL glass beaker to form an oil–water mixture. The aerogel specimen was placed at the oil–water interface, and the removal process was monitored over 5 min. Subsequently, the aerogel was collected, and the remaining oil on the water surface was examined.

### 4.7. Statistical Analysis

For statistical analysis, three independently prepared specimens (n=3) were used for each formulation, and results are expressed as mean ± standard deviation (SD). The effects of GWF content, PVA content, and their interaction on bulk density, calculated porosity, water contact angle (WCA), and maximum crude-oil absorption capacity ($Q_{max}$) were evaluated using two-way ANOVA followed by Tukey’s HSD post hoc test where appropriate. For oil absorption kinetics at a fixed PVA content of 0.25 wt.%, differences among GWF levels at 300 s were analyzed using one-way ANOVA followed by Tukey’s HSD test. Statistical significance was considered at p<0.05. This factorial analysis was used to quantitatively assess composition-dependent property changes across the GWF/PVA formulation matrix [69].

## Figures and Tables

**Table 1:** Composition, physical properties, water contact angles, thermal conductivities, and oil absorption capacity of GWF aerogels.

Sample Name	GWF Content (wt.%)	PVA Content (wt.%)	Density (g/cm^3^)	Porosity (%)	Water Contact Angle (°)	Thermal Conductivity (mW/m·K)	Oil Absorption Capacity Q_max_ (g/g)
G1	1.0	0.1	0.014 ± 0.001	99.39 ± 0.03	133.7 ± 2.10	32.1 ± 0.1	43.97 ± 4.22
G2	1.0	0.25	0.016 ± 0.001	99.21 ± 0.01	134.0 ± 2.84	34.1 ± 0.1	55.12 ± 1.57
G3	1.0	0.5	0.020 ± 0.001	98.92 ± 0.03	132.5 ± 1.17	33.3 ± 0.1	33.53 ± 1.51
G4	1.0	1.0	0.025 ± 0.004	98.42 ± 0.23	131.3 ± 1.38	33.4 ± 0.1	43.64 ± 3.47
G5	2.0	0.1	0.024 ± 0.002	98.97 ± 0.07	139.6 ± 2.49	35.8 ± 0.8	31.29 ± 1.58
G6	2.0	0.25	0.027 ± 0.001	98.80 ± 0.06	138.7 ± 0.93	38.0 ± 0.2	34.58 ± 1.80
G7	2.0	0.5	0.028 ± 0.002	98.66 ± 0.09	135.7 ± 1.45	38.5 ± 0.1	32.34 ± 1.46
G8	2.0	1.0	0.034 ± 0.002	98.17 ± 0.12	131.0 ± 0.61	38.2 ± 0.1	28.01 ± 1.02
G9	3.0	0.1	0.036 ± 0.002	98.49 ± 0.10	141.3 ± 1.61	38.2 ± 0.1	25.23 ± 3.54
G10	3.0	0.25	0.038 ± 0.001	98.34 ± 0.06	133.1 ± 3.06	33.2 ± 0.1	22.36 ± 2.36
G11	3.0	0.5	0.041 ± 0.004	98.12 ± 0.22	133.9 ± 2.45	33.3 ± 0.1	20.18 ± 0.92
G12	3.0	1.0	0.046 ± 0.004	97.68 ± 0.21	139.4 ± 1.08	33.2 ± 0.1	18.86 ± 1.50

**Table 2:** Incremental crude-oil uptake capacities and oil-recovery efficiencies of MTMS-modified GWF aerogels over consecutive absorption–mechanical squeezing cycles.

Sample Name	ΔQ_1_ (g/g)	ΔQ_2_ (g/g)	ΔQ_3_ (g/g)	ΔQ_4_ (g/g)	$\eta_{1}$ (%)	$\eta_{2}$ (%)	$\eta_{3}$ (%)	$\eta_{4}$ (%)
G1	43.97 ± 4.22	N.D.	N.D.	N.D.	N.D.	N.D.	N.D.	N.D.
G2	55.12 ± 1.57	N.D.	N.D.	N.D.	N.D.	N.D.	N.D.	N.D.
G3	33.53 ± 1.51	23.37 ± 2.49	18.66 ± 1.49	16.55 ± 4.54	88.88	89.71	88.16	85.58
G4	43.64 ± 3.47	27.68 ± 5.84	24.44 ± 4.61	24.72 ± 6.38	88.60	89.61	88.13	88.71
G5	31.29 ± 1.58	15.82 ± 1.36	12.60 ± 2.49	10.37 ± 0.97	89.19	87.69	85.74	84.09
G6	34.58 ± 1.80	17.15 ± 1.83	14.08 ± 1.23	11.74 ± 3.84	91.83	90.07	88.19	86.06
G7	32.34 ± 1.46	16.91 ± 1.92	13.56 ± 1.07	12.05 ± 1.04	93.10	88.81	87.10	86.79
G8	28.01 ± 1.02	20.55 ± 0.81	17.04 ± 2.85	16.85 ± 2.66	89.70	89.56	87.92	87.89
G9	25.23 ± 3.54	12.54 ± 0.59	10.85 ± 0.82	8.37 ± 1.03	90.05	85.39	84.98	82.13
G10	22.36 ± 2.36	13.97 ± 1.55	12.83 ± 0.18	10.55 ± 3.39	87.94	85.99	85.73	83.67
G11	20.18 ± 0.92	10.12 ± 0.18	8.74 ± 0.33	8.29 ± 0.78	89.11	84.81	82.70	82.27
G12	18.86 ± 1.50	12.35 ± 4.30	10.53 ± 4.38	8.33 ± 1.79	89.32	86.51	84.64	82.96

Note: N.D., not determined because G1 and G2 lost structural integrity during the first mechanical recovery step. Samples G3–G12 lost structural integrity after the fourth recovery step.

**Table 3:** Experimental equilibrium capacities and kinetic parameters obtained from linearized and nonlinear fitting of the pseudo-first-order (PFO) and pseudo-second-order (PSO) models, together with Weber–Morris-type analysis, for selected MTMS-modified GWF aerogels.

Kinetic Parameter	1 wt.% GWF	2 wt.% GWF	3 wt.% GWF
Experimental equilibrium oil absorption capacity Q_e,exp_(g/g)	54.89 ± 1.90	34.51 ± 2.90	22.23 ± 0.68
Linearized PFO model			
R^2^	0.9047	0.9732	0.8354
k_1_ (s^–1^)	0.0127	0.0157	0.0122
Linearized PSO model			
Calculated equilibrium oil absorption capacity, Q_e,cal_(g/g)	55.29	35.16	22.43
R^2^	0.9999	0.9991	0.9998
k_2_ (g·g^−1^·s^−1^)	0.0068	0.0040	0.0130
Nonlinear PFO model			
Q_e,cal_(g/g)	53.49	32.41	21.52
R^2^	0.4290	0.4201	0.3132
RMSE (g/g)	1.205	1.639	0.547
$\mathrm{SSE}\;(({g/g)}^{2}$)	11.611	21.499	2.391
Nonlinear PSO model			
Q_e,cal_(g/g)	54.15	33.34	21.77
R^2^	0.7191	0.6858	0.5784
RMSE (g/g)	0.845	1.207	0.428
$\mathrm{SSE}\;(({g/g)}^{2}$)	5.712	11.647	1.468
Weber–Morris-type analysis			
$k_{id}$$((g/g)\cdot s^{-1/2}$)	0.340	0.462	0.142
C(g/g)	49.56	27.06	19.90
R^2^	0.9542	0.9663	0.9757

Note: RMSE, root-mean-square error; SSE, sum of squared errors.

## Data Availability

The data presented in this study are available from the corresponding author upon reasonable request.

## Abbreviations

The following abbreviations are used in this manuscript:

MTMS	Methyltrimethoxysilane
PVA	Poly(vinyl alcohol)
GWF	Glass wool fiber
SEM	Scanning electron microscopy
FTIR	Fourier-transform infrared spectroscopy
XRD	X-ray diffraction
WCA	Water contact angle
PFO	Pseudo-first-order
PSO	Pseudo-second-order
ANOVA	Analysis of variance
HSD	Honestly significant difference
RMSE	Root-mean-square error
SSE	Sum of squared errors
